# Conservation in first introns is positively associated with the number of exons within genes and the presence of regulatory epigenetic signals

**DOI:** 10.1186/1471-2164-15-526

**Published:** 2014-06-26

**Authors:** Seung Gu Park, Sridhar Hannenhalli, Sun Shim Choi

**Affiliations:** Department of Cell Biology and Molecular Genetics, Center for Bioinformatics and Computational Biology, University of Maryland, College Park, Maryland, MD 20742 USA; Department of Medical Biotechnology, College of Biomedical Science, and Institute of Bioscience & Biotechnology, Kangwon National University, Chuncheon, 200-701 South Korea

## Abstract

**Background:**

Genomes of higher eukaryotes have surprisingly long first introns and in some cases, the first introns have been shown to have higher conservation relative to other introns. However, the functional relevance of conserved regions in the first introns is poorly understood. Leveraging the recent ENCODE data, here we assess potential regulatory roles of conserved regions in the first intron of human genes.

**Results:**

We first show that relative to other downstream introns, the first introns are enriched for blocks of highly conserved sequences. We also found that the first introns are enriched for several chromatin marks indicative of active regulatory regions and this enrichment of regulatory marks is correlated with enrichment of conserved blocks in the first intron; the enrichments of conservation and regulatory marks in first intron are not entirely explained by a general, albeit variable, bias for certain marks toward the 5’ end of introns. Interestingly, conservation as well as proportions of active regulatory chromatin marks in the first intron of a gene correlates positively with the numbers of exons in the gene but the correlation is significantly weakened in second introns and negligible beyond the second intron. The first intron conservation is also positively correlated with the gene’s expression level in several human tissues. Finally, a gene-wise analysis shows significant enrichments of active chromatin marks in conserved regions of first introns, relative to the conserved regions in other introns of the same gene.

**Conclusions:**

Taken together, our analyses strongly suggest that first introns are enriched for active transcriptional regulatory signals under purifying selection.

**Electronic supplementary material:**

The online version of this article (doi:10.1186/1471-2164-15-526) contains supplementary material, which is available to authorized users.

## Background

Recent complete sequencing projects have confirmed that almost all eukaryotes have introns [1–6]. Different species harbor dramatically different density and length of introns, ranging from a few bps to hundreds kbps [5, 7, 8]. Generally speaking, genes in higher eukaryotes such as mammals have a greater number of introns than those of lower eukaryotes such as yeast, *Drosophila*, and *C. elegans* [5, 7, 8]. These differences may partly be explained by the differences in modes of intron removal between lower and higher eukaryotes [9], as well as differences in selective pressure. A substantial fraction of introns in the human genome have likely originated early in eukaryotic evolution, and dynamic evolutionary changes such as intron gain and loss have structured the eukaryotic introns since [10–14]. Noticeably, the number of genes is relatively stable across organisms from *C. elegans* (~19,000) and *Drosophila* (~14,000) to humans (~25,000), while the fraction of non-coding DNA including introns greatly varies up to several folds [15, 16], some of which is likely to underlie species-specific adaptations [17, 18].

The mere existence of introns in the eukaryotic genome is intriguing, given the cost of transcription and the cost of maintaining a splicing regulatory system that ultimately eliminates the introns from the functional product of the gene. In particular, whether introns have evolved under selective constraints, and the extent thereof, are not entirely clear; while some studies suggest that introns evolve largely free from selective constraints [19–22], others imply that intron sequences are subject to considerable levels of evolutionary constraints [22–25]. Using intronic multispecies conserved sequences (MCSs), Sironi et al. [26] showed that the MCS density steadily increase with intron length with MCSs occupying up to 10% of total size in long introns, and also that MCSs are enriched in genes involved in development and transcription. Based on 225 intron fragments in *D. melanogaster* and *D. simulans,* Haddrill et al. [27] demonstrated that a substantial portion of intronic sites is likely to be evolving under considerable selective constraint and this tendency increases with intron length. Furthermore, Vinogradov [28] showed that the length of conserved intronic sequence in the human genome is greater in proteins with the larger numbers of functional domains. Even though several reports have shown an enrichment of conserved sequences in introns, in various species [23–25, 28, 29], these claims are not without controversies. In one instance, it was shown that intronic sequences evolve faster than fourfold degenerate sites when splicing regulatory sequences were excluded [22]. These discrepancies can be partly ascribed to biases in the data sets with different ranges of lengths of introns studied [27]. Besides their obvious role in isoform regulation, introns have also been shown to harbor regulatory signals and noncoding genes [30–33]. Thus, overall, it is highly likely that portions of intronic sequences are evolving under selective constraints consistent with their functional importance.

The 5’-most “first” intron differs from the other introns in several aspects in terms of processing, epigenomic marks, length and evolution. First, in terms of processing, despite an overall 5’-to-3’ trend in splicing during transcription, the first intron is removed (on average) somewhat later [34]. Second, the 5’ end of the first intron displays a specific epigenomic context being enriched for two activating histone modifications H3K4me3 and H3K9ac [35]. Finally, the “first” introns have a special status, as these are typically the longest among all introns and appear to be most selectively constrained. More conserved blocks were found in first introns relative to other introns in several species [36]. Consistently, some studies have shown that intron divergence has a significant negative correlation with the length of first introns in *Drosophila* [27, 37]. Zheng et al. [38] have also reported that, in *Tetrahymena*, the most conserved introns are found closer to the 5’ end of genes. Furthermore, introns harboring regulatory elements tend to be the first introns [38–40], and, in fact, the frequencies of certain regulatory motifs are greater in first introns [41]. Overall, it seems that, among the various possible roles of introns, first introns have especially evolved to harbor regulatory elements.

Previous investigations of potential regulatory role of first intron were mainly performed in Drosophila or plants. The extent to which the previous conclusions hold true in mammals is not known. Moreover, the recent explosion of human epigenomic data via the ENCODE project (Table 1) provides a unique opportunity to investigate regulatory potential of first introns and its correlates thereof in human. Based on human RefSeq gene annotation from UCSC and 46 vertebrate species conservation (including primate and mammal subsets) (Table 1), we first show that blocks of highly conserved sequences are significantly enriched in first introns relative to other introns. Using genome-wide profiles of several epigenomic marks from the ENCODE database, we show that the first introns are also enriched for chromatin marks indicative of active regulatory regions in a manner consistent with conserved blocks. Interestingly, conservation in first intron as well as active chromatin marks of a gene correlate positively with the numbers of exons in the gene. While these correlations also hold true for conservation and active marks in gene’s upstream region, they are significantly weaker in second introns and negligible beyond the second intron. In summary, our results strongly suggest that first introns in human are enriched for evolutionarily selected active transcriptional regulatory signals that are likely to be important for regulating complex gene expression patterns of large multi-domain genes.

**Table 1:** **List of data resources**

Resources	URLs
**ENCODE**	http://www.nature.com/encode/#/threads
**UCSC chromosome sequence**	http://hgdownload.soe.ucsc.edu/goldenPath/hg19/chromosomes/
**46 species multiple alignment**	http://hgdownload.cse.ucsc.edu/goldenpath/hg19/multiz46way/alignments/
**The Genome Reference Consortium**	http://www.ncbi.nlm.nih.gov/projects/genome/assembly/grc
**PhastCons**	http://hgdownload.cse.ucsc.edu/goldenPath/hg19/phastCons46way/
**DNaseI Hypersensitivity Uniform Peaks from ENCODE/Analysis**	http://genome.ucsc.edu/cgi-bin/hgFileUi?db=hg19&g=wgEncodeAwgDnaseUniform
**Transcription Factor ChIP-seq Uniform Peaks from ENCODE/Analysis**	http://genome.ucsc.edu/cgi-bin/hgFileUi?db=hg19&g=wgEncodeAwgTfbsUniform
**Histone Modifications by ChIP-seq from ENCODE/Broad Institute**	http://genome.ucsc.edu/cgi-bin/hgFileUi?db=hg19&g=wgEncodeBroadHistone
**RNA-Seq Atlas**	http://medicalgenomics.org/rna_seq_atlas/download

## Results

### First introns are the most conserved

As a proxy for purifying selection, we compared evolutionary conservation across introns grouped by their position in the transcript structure. Conservation in an intron was estimated as the fraction of intron sites that were conserved based on a PhastCons score threshold. Three different multiple alignments (primates, mammals and vertebrates) and different PhastCons score thresholds were used. Our conclusions hold for all choices of alignment and threshold; here we only present the results based on mammalian conservation with PhastCons score threshold of 0.5. The primary focus of our investigation was transcriptional regulatory elements in introns. Therefore, to exclude the possibility of splice site signals biasing the conservation score, especially for short introns, the sequences within 300 bps of the splice junction, which are considered to harbor splicing regulation signals [42], were excluded from our analysis. Based on these criteria, the median conservation per intron was only 2.1%. Introns were grouped by their positions from the 5’ end of the transcript; for instance, all first introns were in the first group. The fraction of conserved sites was then estimated within each group.

**Figure 1:**
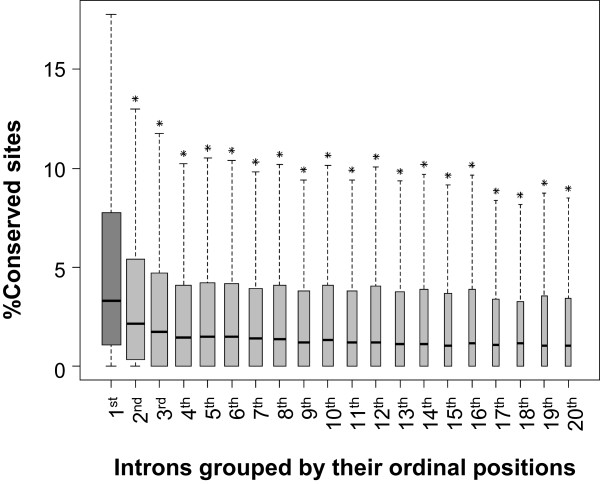
**Sequence conservation in intron ordinal groups.** Introns were grouped by their ordinal positions. Introns containing repeats were removed and for each remaining intron 300 bps from the 5’ as well as the 3’ end were removed to minimize interference from splicing signals (see Methods). Box plot analysis is performed for the proportions of conserved sites in introns grouped by ordinal positions from 1^st^ introns to 20^th^ introns. The proportions of conserved sites in first introns are represented by darker gray colors than those in the other downstream introns. The figure shows that first introns have the highest proportions of conserved sites and the proportion decreases monotonically with increasing ordinal number, stabilizing at 4^th^ intron group. ‘*’ indicates p < 2.2e-16. Note that fewer introns are collected from the higher ordinal positions indicated by narrower box width.

We found that the median conservation in first introns (3.3%) was significantly higher than all other groups using Wilcoxon rank sum test (p < 2.2e-16) (Figure 1). A potential bias in this analysis stems from the fact that shorter transcripts with few introns are more abundant relative to long genes with several introns and will therefore dominate the first intron group. To avoid this potential bias we repeated the analysis with different sets of transcripts grouped by number of introns, i.e., transcripts were first segregated based on the number of introns, and the analysis was repeated separately for all the groups, as illustrated in Additional file 1: Figure S1A. As shown in Additional file 1: Figure S1B, a similar trend was observed: first introns are the most conserved.

### Chromatin signals are the highest in first introns and increase with increasing conservation

Conserved regions in first intron are likely to play a transcriptional regulatory role as suggested by previous studies [43–51]. Several chromatin marks have been shown to associate with transcriptional regulatory regions [52–54]. Next we assessed whether the first introns are enriched for specific regulatory signals, similar to the conservation analysis. We obtained a number of chromatin marks and protein-binding data from ENCODE [55, 56] for three cell lines - GM12878, H1-hESC, and K562. The following data were included: DNase I hypersensitivity sites (DHS), Transcription factor binding sites (TFBSs) for 80 TFs in GM12878, 50 TFs in H1-hESC, and 112 TFs in K562, active chromatin marks (e.g., H3K4me1, H3K4me3), a repressive chromatin mark (e.g., H3K9me3), and the insulator protein CTCF binding sites.

**Figure 2:**
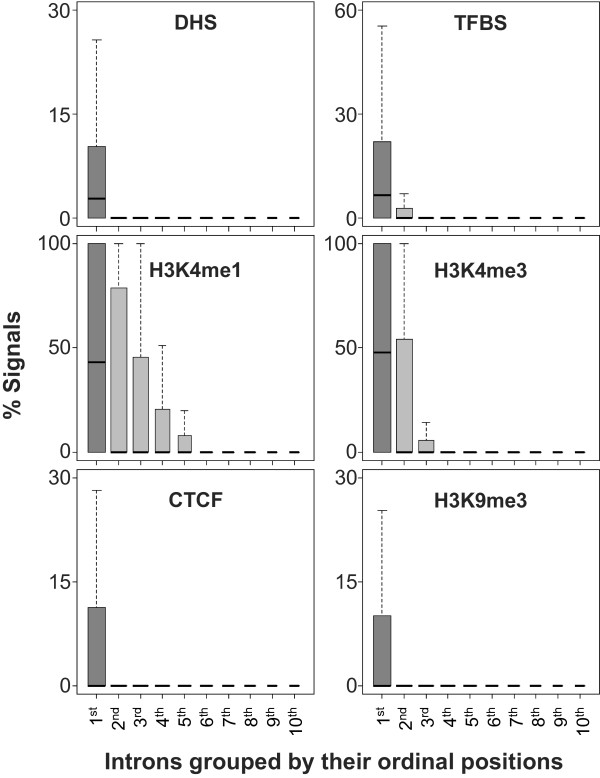
**Proportions of regulatory chromatin marks in intron ordinal groups.** All the signals are derived from GM12878 cell line. Using the peak values for each signal, box plot analysis is performed for the proportions of the chromatin marks sites in introns from each ordinal group are estimated. Results of the same analyses in the two other cell lines are presented in Additional file 1: Figure S2A, B. The proportions of the peak signals of each chromatin regulatory marks in first introns are represented by darker gray colors than those in the other downstream introns. As shown, the proportions of the regulatory chromatin marks are found to be the highest in first introns compared to the other downstream introns.

**Figure 3:**
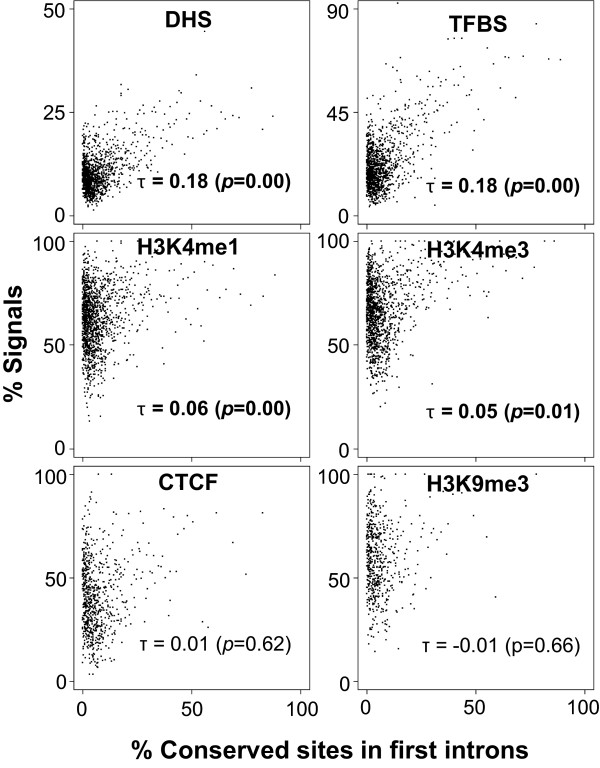
**Correlation between regulatory signals and conservation in first introns.** Kendall’s tau correlation analysis is performed to test how the conservation in first introns is related to density of regulatory marks. For smoothing, introns are binned into groups of 10 genes by conservation and average regulatory signal density is calculated for each bin, and plotted against the average conservation of the group. As in Figure 2, all marks are obtained from GM12878 cell line, and the results from the other two cell lines are provided in Additional file 1: Figure S3A, B. Kendall’s tau values and *p*-values are shown; significant *p*-values (*p* < 0.05) are represented by bold font. All active chromatin marks show significant positive correlations between conservation and the proportions of the regulatory marks.

Figure 2 shows the regulatory signals in different intron groups. The results in different cell lines were similar and here we show the results based on data from GM12878 while the results based on other cell lines are provided as Supplementary Figures. Our analysis shows a clear enrichment of epigenomic signals in first introns relative to other introns (Figure 2 and Additional file 1: Figure S2A-B). Next we assessed whether enrichment of epigenomic signals in introns correlated with that for conservation. As shown in Figure 3 and Additional file 1: Figure S3A-B, we found this to be generally the case, significant (p < 0.01), the correlations are relatively modest (τ ~ 0.2), specifically for active chromatin marks, DHS, and TFBS. However, there was weak or no correlation for repressive chromatin marks such as H3K9me3 and CTCF binding sites. These trends are consistent in the two other cell types, hESC and K562 (Additional file 1: Figure S2A-B).

Next we contrasted the above conservation results for first introns with that for proximal promoters of the genes, known to harbor conserved regulatory signals [57–61]. We found that the correlations between conservation and epigenomic marks also hold for 2 kb proximal promoter regions of the gene (Additional file 1: Figure S4A-C), which suggests an enrichment of conserved regulatory signals specifically in first introns, akin to proximal promoters.

### First intron conservation and regulatory signals positively correlate with the numbers of exons

**Figure 4:**
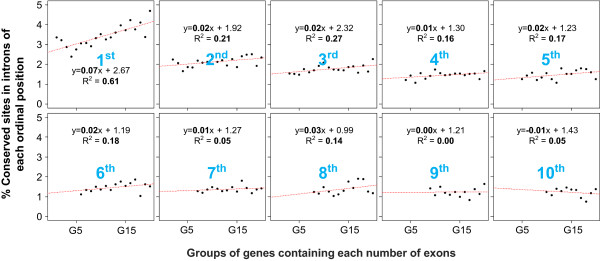
**Relationship between intron conservation and the numbers of exons.** Linear regression analysis is performed to see the relationship between the degree of conservation in introns from each ordinal position and the numbers of exons within genes. Genes are grouped by the numbers of exons within genes. For example, as shown in the top left box in the figure, genes with two exons are grouped together (named G1), the average degrees of conservations in first introns of the genes in G1 in X-axis is shown on the Y-axis. As for G1, the conservations in first introns in genes with three exons (named G2) and up to genes with twenty-one exons (named G20) are calculated. Likewise, in the box for 2^nd^ introns (shown in blue), genes are grouped as in the first box but now the conservation in second intron is estimated; likewise for introns 3 up to 10. Note that the numbers of dots decreases by one in each subsequent box, because N^th^ (N>=1) introns are non-existent in genes comprising less than N numbers of exons. Regression equations and R-squared values for each linear regression analysis are shown. The collection of plots suggests that there is strong correlation between first intron conservation and number of exons, specifically for the first intron, and much lesser extent for other introns.

Given that mammalian first introns can often be very long and thus harbor numerous conserved, potentially regulatory regions, we assessed the relationship between level of conservation and the complexity of gene’s expression. From an information theoretic perspective, expression complexity is defined in terms of the number of tissues (or conditions) in which a gene is expressed. Genes that expressed in a very few or most tissues have low complexity whereas the genes expressed in an intermediate number of tissues have the highest expression complexity [28, 62]. It was shown that expression complexity is positively correlated with both coding and intronic content of the gene [28, 62]. Therefore we used number of exons as a proxy for gene expression complexity. We then estimated the correlation between the first intron conservation and number of exons for all genes. Interestingly, we found striking positive correlations between conservation in first intron and the numbers of exons (Figure 4). A similar but weak positive trend was found in the case of conservations in second introns but the trend greatly weakened after second intron (Figure 4). We compared the results for first introns with that for the 2 kb proximal promoter region. As expected, the promoter conservations correlated positively with the numbers of exons (Additional file 1: Figure S5). However, when the same analysis was performed for 2 kb downstream flanking regions of genes, as shown in Additional file 1: Figure S5, the trend was still significant but very weak or non-existent.

**Figure 5:**
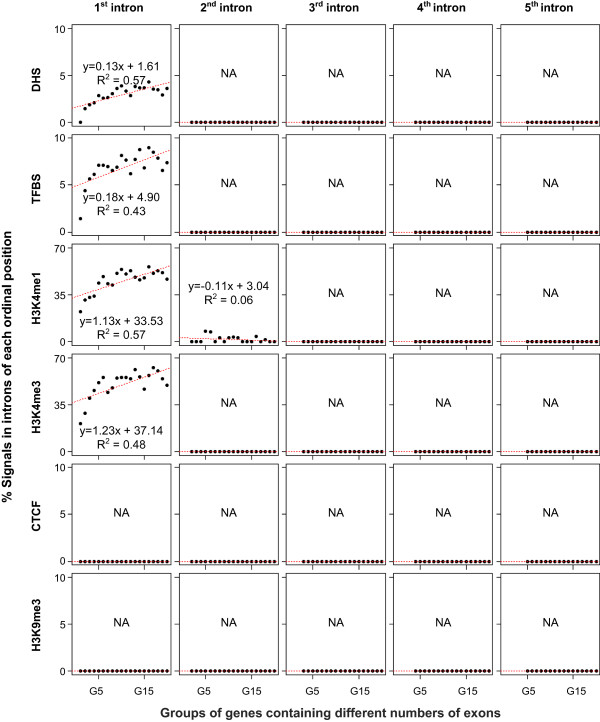
**Relationships between the proportions of regulatory signals in introns and the numbers exons.** Analysis similar to that in Figure 4 is performed but for various regulatory chromatin marks in the introns. Gene groups represented in X-axis are the same as for Figure 4, while the proportions of regulatory marks are used in Y-axis. The figure shows that the proportions of active regulatory chromatin marks in first introns produced the same ascending trend with increasing numbers of exons in genes, and the ascending trend almost disappears from second intron onward, similar to the trend seen for conservation. NA stands for “Not-Assigned” and essentially means that the median values of signals in were 0 and therefore regression could not be performed.

Next we assessed the correlation between the regulatory signals and the number of exons. As shown in Figure 5 and Additional file 1: Figure S6, general regulatory signals (e.g., DHS and TFBS) and active regulatory chromatin marks (e.g., H3K4me1 and H3K4me3) exhibit a significant positive correlation between the regulatory signal proportion and the number of exons, whereas CTCF binding sites and a repressive regulatory chromatin mark, H3K9me3 did not show any such trend. This finding suggests that conservation may have to do with the presence of active regulatory signals rather than of repressive regulatory signals.

### Conservation and active epigenomic marks correlate with level of gene expression

**Figure 6:**
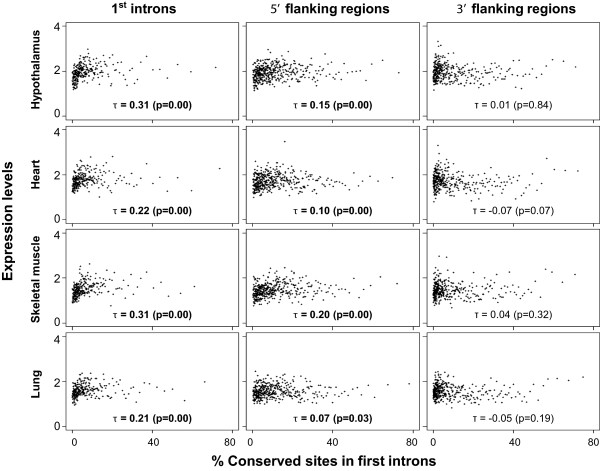
**Relationship between expression levels of genes and the conservation in first intron.** The figure shows the relationship between gene expression level and the first intron conservation for four different human tissues. Then Kendall’s tau correlation test results are shown. Conservations in first intron and upstream flanking region, but not in the downstream region, have significant positive correlations with expression levels of genes. For smoothing, genes are binned into groups of 50 by expression level. Each dot represents the mean values for conservation and the expression levels of 50 genes per bin.

We next investigated whether or not conservation in first introns is related to the level of gene expressions. For this, the degrees of conservation were plotted against the levels of gene expressions in several human tissues. Gene expression data sets were obtained from RNA-seq atlas constructed by [63] from 11 human tissues. Here, we only show a subset of the results from four different tissues including hypothalamus, heart, skeletal muscle, and lung (Figure 6), although the conclusions were the same for all the other tissues (data not shown). Interestingly, conservation is positively correlated with the levels of gene expression in all tissue types. The degrees of conservations in 2 kb proximal promoter also showed the same positive trends (Figure 6), confirming that the conserved sites in first introns and promoters might have something to do with the presence of active regulatory signals rather than repressive signals, consistent with the results of Figure 3. There was no significant positive correlation between the degrees of conservation in 3’ flanking region and expression levels (Figure 6).

Although the analysis thus far was based on all ‘transcripts’, we repeated the analyses by selecting a single representative transcript per gene, resulting in ~16,000 transcripts. As shown in Additional file 1: Figure S7A-D, the analyses at the gene level yield qualitatively the same results.

### Regulatory signals preferentially occur in conserved regions in first introns - a gene-wise assessment

**Figure 7:**
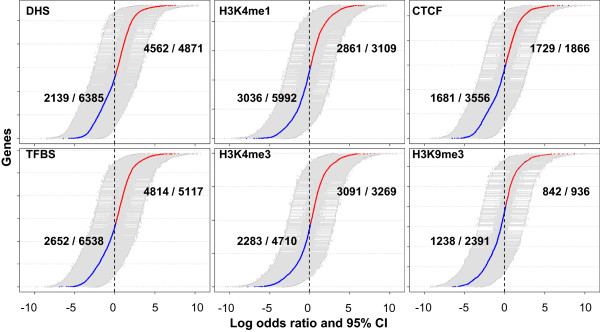
**Enrichment of regulatory signals in conserved portion of first intron relative to non-conserved portion.** After dividing each first intron into two groups, conserved sites and non-conserved sites, log-odds ratios (X-axis) are computed with 95% confidence interval (CI) (light gray bars) for each gene. The log-odds greater than zero are represented by red dots. Each box provides the analysis result done for each regulatory mark. Y axis represents each gene corresponding to each log-odds ratio. The numbers of genes with a statistically significance (p < 0.01) divided by the total numbers of genes used for testing are presented in the middle of each box.

Our analysis thus far is based on grouped data. Next we assessed for each gene whether the regulatory signals in the first intron preferentially occur within conserved region. We partitioned each first intron into conserved and non-conserved regions, obtained percent coverage by regulatory signals in each part and calculated the log-odds ratio with 95% confidence intervals (see Methods) (Figure 7 and Additional file 1: Figure S8). Note that the total numbers of genes in each panel in Figure 7, corresponding to different regulatory marks, are different because of missing data for some genes in each case. As shown in Figure 7, for some of the signals (DHS, TFBS, H3K4me3) there are many more genes with significant enrichment of the signal in conserved regions relative to the genes with significant enrichment in non-conserved regions of the first intron. For some other signals (H3K4me1, H3K9me3) the opposite is true, while for CTCF, there is no clear winner. An enrichment of accessible chromatin and TFBS binding in conserved regions of the first intron is consistent with their role in active gene regulation. We also found that that promoter-specific mark H3K4me3 [54] is also enriched among conserved regions in first introns, while H3K4me1, which is associated with both enhancers and promoters, is not. Also, a lack of enrichment of repressive chromatin mark H3K9me3 in the conserved regions is consistent with the result of Figure 3 showing weak or no correlation between the proportions of the repressive chromatin marks in first introns and the degrees of conservation.

### Trends in the first introns are independent from those in promoters

A substantial portion of transcriptional control of a gene is mediated by signals in its proximal promoters. In fact, numerous ChIP-seq data for TFBS from the ENCODE project reveal a distribution of binding sites around the transcription start site, into the introns [56, 64]. A 5’ enrichment of certain epigenetic marks has also been noted with regards to splicing signals [35].

We first investigated whether the proportions of conservation and epigenetic marks are biased toward the 5’ end of the intron. After excluding the short first introns (shorter than the median length) each intron was binned into five equal-sized bins, and we estimated for each bin, the fraction of introns in which the highest signal in the intron was in the particular bin. As shown in Additional file 1: Figure S9, there is varying enrichment of signals toward the 5’ bins but in absolute terms only in a small fraction of introns the highest signal is in the 5’-most bin. Moreover, this trend is not uniformly observed for all regulatory signals.

Despite an enrichment of signals toward 5’ as well as the fact that the general patterns of enrichment in the first introns also hold for the 5’ flanking regions (Additional file 1: Figure S4 and S5), it is not immediately clear whether the patterns in the two regions are related by virtue of extended conserved and regulatory regions spanning the promoter and the first introns. To ensure that the observed patterns in first introns are not simply due to signals spanning the two regions, we tested if the trends in the first introns are maintained after removing such “spillover” signals. We reasoned that the genes in which a particular signal in first intron is simply due to spillover from the promoter should exhibit a greater proportion of that signal in promoter and first exon relative to the first intron. Thus we excluded the genes in which the proportion of a signal in the first exon and promoter was at least as high as that in the first intron. Despite the reduction in statistical power owing to much reduced dataset, all the trends were still maintained (Additional file 1: Figure S10A-D). Next, to exclude the interference of signals between introns and exons or flanking regions of other genes, we repeated the analyses after excluding the genes whose first intron overlapped with exon or flanks of another gene. As shown in Additional file 1: Figure S11A-D, all trends in the first introns are still maintained after removing the overlapped sets. All these results suggest that the first intron conservations and the enrichment of regulatory signals are independent of the trends for the promoter.

### Trends in the first introns are not due to their proximity to the transcriptional start site

Given that many of the trends in first intron are similar to those in the proximal promoter, next we assessed whether the trends in the first introns are simply due to their proximity to the transcription start site (TSS). Thus we investigated the trends in the first introns compared to that in the second introns when controlled for their distance from the TSS. We categorized first and second introns into bins corresponding to distance of the 5’ end of the intron from the TSS and compared the first and the second intron trends within each bin. Note that because first introns are much longer than the second intron (Additional file 1: Figure S12A) and because we are controlling for the distance to the 5’ end of the intron, in the direct comparison, on average, the distances within first introns is much greater than those in second introns, thus rendering this comparison conservative. As shown in Additional file 1: Figure S12A, distances from the TSS to the first and the second intron are vastly different and overlap only in a short range 500–1000 bps. For various distance bins within in this range, we compared the conservation and chromatin signal proportions between the first intron and the second intron.

**Figure 8:**
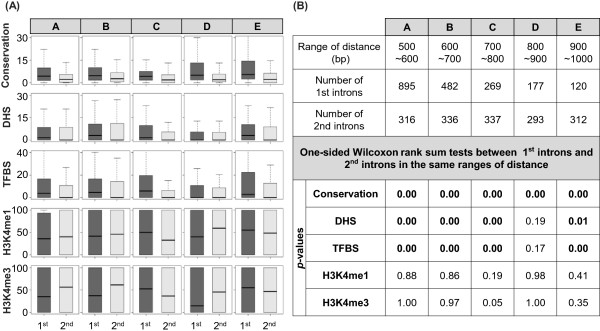
**Comparison of trend in first and second introns after controlling for their distance from the TSS.** Using the start of first exon as a proxy for the TSS, distance from the first intron and the second intron to the TSS was obtained. Additional file 1: Figure S10A shows the length distribution of the two introns. Only the introns whose distance from TSS was in the overlapping range of 500–1000 bps were included in this analysis. Within this distance range, first and second introns were partitioned into smaller distance bins, and within each bin, various marks were compared between the first and the second introns. **(A)** Dark gray and light gray represent the proportions estimated in the first and the second intron respectively. **(B)** Table for the number of genes and corresponding statistics estimated by one-sided Wilcoxon rank sum tests for each comparison illustrated in **(A)**.

As shown in Figure 8, the proportions of conservation, DHS, and TF sites in the first introns were almost always higher than in the second introns in all distance bins, suggesting that the observed trends are a property of first introns rather than of their proximity to the TSS alone. Furthermore, there was no clear difference between first and second introns for H3K4me1 and H3K4me3 signals, and there was no clear increasing or decreasing trends of conservation or signals with increasing the distances to the TSS (Figure 8). The loss of the trends in the two histone marks may partly be due to the breadth of those signals often spanning the entire genic regions, thus introducing error in estimation of precise proportions of those signals, which can overwhelm real signals in a relatively small dataset. Another possible explanation for the loss is cell-type dependency of those signals. As shown in Additional file 1: Figure S12B, the trend of H3K4me1 is still valid for h1 ESC cell line. Repetition of these analyses in other cell types (Additional file 1: Figure S12B, C) shows consistent results, indicating that the proximity of first introns to the TSS is not the main cause for the observed trends in the first introns.

## Discussion

Introns are ultimately removed from protein synthesis as a part of post-transcriptional processing, yet all the intron sequences are respectively copied as pre-mRNAs or DNAs sequence-by-sequence during transcription and replication, which seems to cause huge energetic burden to cell. About 2 ATP is known to be consumed for 1 bp synthesis during transcription [65]. Considering that introns can be as long as hundreds of kilobases, their maintenance must entail a substantial cost to the cells. Nevertheless, most eukaryotic genes have introns [1–6], and although introns can be lost, in general, introns have been maintained during eukaryotic lineage evolution [5, 6, 66, 67]. Evolutionary maintenance of introns despite the energetic burden they entail suggests some evolutionary advantage afforded by introns.

Several studies have presented various possible scenarios for how introns provide advantage to cell’s survival [68–72]. It is also true that, for a long time, introns have been considered to be non-essential for the most part [19–21]. In fact, approximately 3% of genes in the human genome are intronless genes [73]. Furthermore, no significant functional changes have been detected in many experimental designs with or without introns for the same coding sequences [74]; however this counterintuitive finding may be simply due to the fact that in molecular biology experiments, ‘gene function’ generally has been equated to ‘protein function’, devoid of its regulatory context.

Transcriptional regulatory signals encoded within introns represent one of the main selective advantages afforded by introns [32]. Two types of regulatory signals have been reported in introns; classical enhancers and intron-mediated enhancers (IME). Several classical enhancers, i.e., *cis*-regulatory elements regulating spatio- temporal gene expression, are known to locate within introns in mouse transgenic experiments [75], for instance, the enhancer elements of GLI3, an important transcription factor of Sonic hedgehog signaling [76]. In contrast, IME suggest a broad role of an intron, first intron in particular, in regulating expression level, without ascribing the function to a specific region within the intron, suggesting a mechanism different from that for classical enhancers [32, 77]. For instance, in experiments performed in Arabidopsis, rice, as well as mammal, the expression level of a gene with intron, particularly first intron, could increase up to 100-fold compared to the expression construct with the same coding sequences but without the introns [78]. IME activity was found to be dependent on the location and distance from transcription start site unlike the classical enhancers [79]. Whether or not IME is a general mechanism of expression control is not known.

Here, we have investigated the functional importance of first introns in human by quantifying their evolutionary conservation and potential regulatory content relative to other introns. Sequence conservation is considered to reflect the resistance against random mutations through purifying selection. Identifying conserved regions in genomes has thus been one of primary criterion to detect functional regions of genomes. Previous studies in several species including *Drosophila* and *Arabidopsis* [27, 37, 41, 80] have shown that first introns tend to be the longest and the most conserved, which is recapitulated in human by our study (Figure 1). We further investigated the reasons of higher conservations in first introns by testing their association with various regulatory marks, and the associations of conservation and regulatory signals in first introns with the gene’s expression level (Figures 2, 3, 4, 5 and 6). This analysis also underscores the importance of epigenomic data, which became available only recently by ENCODE project (Table 1), in interpreting the function of the non-coding portion of the genome. One of the interesting findings of this work is that genes with higher density of conservation and active regulatory marks but not repressive marks in first introns tend to have more exons that encode longer proteins (Figures 4 and 5), which can be interpreted to suggest that long functionally complex proteins may also be under a richer regulatory control. It is not entirely clear why only active regulatory signals but not repressive signals have positive correlations with conservation and number of exons. It may partly be due to the tendency of repressive signals to be broad and less intense relative to activating signals [64], which can result in lower discrimination of a specific region as well as lower detectability and lower statistical power. Overall, by leveraging the recent explosion in epigenomic data, our work lends further support, particularly in human, to the notion that introns, and especially the first introns, harbor evolutionarily constrained regulatory regions mediating both the level and complexity of gene expression. However several important questions remain open.

In our gene-specific analysis of enrichment of various regulatory signals in the conserved portion of the first intron, we found that in first introns, the conserved regions are favored by DHS, TFBS binding, and H3K4me3, which suggests that the conserved region may have a role in active gene regulation. More interestingly, we found a difference in two activating marks – H3K4me1 and H3K4me3. While both these marks are associated with proximal promoters, only H3K4me1 is associated with distal enhancers [54]. This subtle difference in enrichment may suggest that the conserved regions in the first intron are more promoter-like and less like a distal enhancer in their mode of action. However, this effect is not simply due to spillover of promoter signals into enhancer as we showed above (Additional file 1: Figure S10A-D). Further mechanistic disambiguation of this difference will require additional studies.

## Conclusions

In the present study, we investigated the potential regulatory role of first introns in human genes by leveraging the recent explosion in epigenomic data by the ENCODE project. In addition to extending the previous results in Drosophila and plant to human, i.e. showing that the first introns are enriched for conserved regions, we show that these higher conservations in first introns are related to 1) the presence of active regulatory chromatin marks, 2) higher expression levels of genes, and 3) a greater number of exons within genes. Overall, our results strongly suggest that first introns in human are enriched for evolutionarily selected active transcriptional regulatory signals that are likely to be important for regulating complex gene expression patterns of large multi-domain genes.

The precise mechanism by which individual conserved, putative regulatory regions in the first intron, regulate gene expression, as well as other potential functions of conserved regions in the first intron, are unclear. The extreme lengths of mammalian first introns represent another enigma. The evolutionary path leading up to long introns as well as whether the first intron length is under selective constraint are not known. Finally, whether it is beneficial for the regulatory elements to reside within the first intron, as opposed to, say, the upstream region of the gene, or whether evolution is neutral to this outside-inside choice, is another open and interesting question. To ultimately resolve the mystery of introns, these are some of the questions that will need to be addressed.

## Methods

### Obtaining of introns, 5’-, and 3’-flanking regions from the human genome

The exon-intron position information of 36,024 Refseq mRNA (KnownGenes) without duplicates was downloaded from the UCSC Table Browser (January 2013). Genomic sequences for each chromosome were obtained from the primary GRCh37/hg19 assembly, and were used for retrieve intron sequences. Very short introns (less the 1 kb in length) were excluded, as well as very long introns (greater than (third quartile + (interquartile range × 1.5)) in length to minimize the outlier effects. To minimize interference from splicing signals in interpreting intronic conservation, we excluded 300 bps from both the 5’ and 3’ ends of each intron [42]. Then repeat elements were masked by RepeatMasker [81]. The numbers for introns in each ordinal position group after all these filtrations are shown in Figure 1. Additionally, we obtained the 2 kb region upstream of the first exon and 2 kb region downstream of the last exon, as a proxy for 5’ and 3’ flanks of genes.

### Estimating conservation

PhastCons scores were used to estimate position-wise sequence conservation [82]. The PhastCons scores for 46 placental mammal subset were downloaded from the UCSC genome browser (Table 1, March 2013). The Phastcons scores were overlaid onto the intron regions and 5’/3’-flanking regions obtained by the methods described above section. Sites with PhastCons score ≥ 0.5 were considered as ‘Conserved’ sites. The proportions of conserved sites were then estimated for each group of introns grouped by their ordinal positions, and 5’/3’-flanking regions. Conservation in first intron for each gene was estimated by the number of conserved sites divided by the total length of the intron.

### Obtaining and mapping regulatory signals and chromatin marks

Genome positions of peaks (region of statistically significant signal enrichment) for DHSs, TFBSs and chromatin marks measured in the ENCODE Tier-1 cell lines (GM12878, H1-hESC and K562) were downloaded from the ENCODE Project from UCSC genome browser (March 2013). The specific download links are provided in Table 1. The processed peak regions of all of these regulatory signals were extracted, and mapped onto the filtered introns and flanking regions. For each intron and flanking region, we then estimated the proportion of the region covered by each regulatory signal and chromatin mark.

### Obtaining mRNA expression levels estimated by RNA-seq analysis

Krupp et al. [63] have reported the mRNA expression levels in RPKM. We used Log_2_ (RPKM + 1) to report expression levels of mRNAs for 11 different human tissues (Table 1). A total of 32,384 mRNAs have their expression values.

### Statistical tests

Kendall’s tau rank correlation coefficients were computed using R studio (Racine 2012) among the variables representing evolutionary conservations of introns or flanking regions, regulatory signals mapped onto introns or flanking regions, and mRNA expression levels. Introns or flanking regions with no conservation or regulatory signal were excluded; we ascertained that this only makes our results more stringent because the fraction of first introns that were eliminated were smaller than those for other introns and including this with “0” value would make the results even stronger.

To test whether the regulatory signals and chromatin marks are enriched in conserved regions compared to non-conserved regions in each gene, odds ratio of the signal estimates between the two regions was estimated. Briefly, first introns were divided into two regions, conserved first introns and non-conserved first introns by the criteria of Phastcons ≥ 0.5, and the chromatin signals and regulatory signals described in the main text were overlaid into each region, generating a 2×2 contingency table. Odds ratios were then estimated for each gene from the contingency table. The odds ratio is the ratio of the proportion of regulatory signals in conserved sequences to that in non-conserved sequences. The odds ratios were thus computed using the “Text::NSP::Neasures::2D::odds” Perl module, and the 95% confidence intervals associated with odds ratios were calculated using the formulas,  (SC: Signal peaks on conserved sites, NC: Non-signals peaks on conserved sites, SN: Signal peaks on non-conserved sites, NN: Non-signal peaks on non-conserved sites) [83].

## Electronic supplementary material

Additional file 1: Figure S1: Comparison of conservations in first introns with those in the other introns using an alternative grouping strategy. **Figure S2.** Proportions of regulatory chromatin marks in intron ordinal groups in H1-hESC and K562. **Figure S3.** Correlation between regulatory signals and conservation in first introns in H1-hESC and K562. **Figure S4.** Correlation between regulatory signals and conservation in the upstream flanking regions in three different cell lines. **Figure S5.** Relationship between flanking region conservation and the numbers of exons. **Figure S6.** Relationship between the proportions of regulatory signals in introns of each ordinal position and the numbers of exons. **Figure S7.** Analysis based on a single representative transcript for each gene. **Figure S8.** Enrichment of regulatory marks in the first intron in two additional cell lines. **Figure S9.** Five prime to three prime biases in signal density along the first intron. **Figure S10.** Excluding spillover of signals s from the promoter. **Figure S11.** Excluding genes whose first introns overlapped with exons or flanks of another genes. **Figure S12.** Analyzing the effect of proximity to the TSS. (PPTX 936 KB)
