# Supplementary material for: Conservation in first introns is positively associated with the number of exons within genes and the presence of regulatory epigenetic signals
Source: BMC Genomics. 2014 Jun 26;15(1):526. doi: 10.1186/1471-2164-15-526 (PMC4085337; doi:10.1186/1471-2164-15-526)
Supplement: Supplementary file 1 — Additional file 1: Figure S1: Comparison of conservations in first introns with those in the other introns using an alternative grouping strategy. Figure S2. Proportions of regulatory chromatin marks in intron ordinal groups in H1-hESC and K562. Figure S3. Correlation between regulatory signals and conservation in first introns in H1-hESC and K562. Figure S4. Correlation between regulatory signals and conservation in the upstream flanking regions in three different cell lines. Figure S5. Relationship between flanking region conservation and the numbers of exons. Figure S6. Relationship between the proportions of regulatory signals in introns of each ordinal position and the numbers of exons. Figure S7. Analysis based on a single representative transcript for each gene. Figure S8. Enrichment of regulatory marks in the first intron in two additional cell lines. Figure S9. Five prime to three prime biases in signal density along the first intron. Figure S10. Excluding spillover of signals s from the promoter. Figure S11. Excluding genes whose first introns overlapped with exons or flanks of another genes. Figure S12. Analyzing the effect of proximity to the TSS. (PPTX 936 KB) [file 12864_2014_6202_MOESM1_ESM.pptx]

## Slide 1
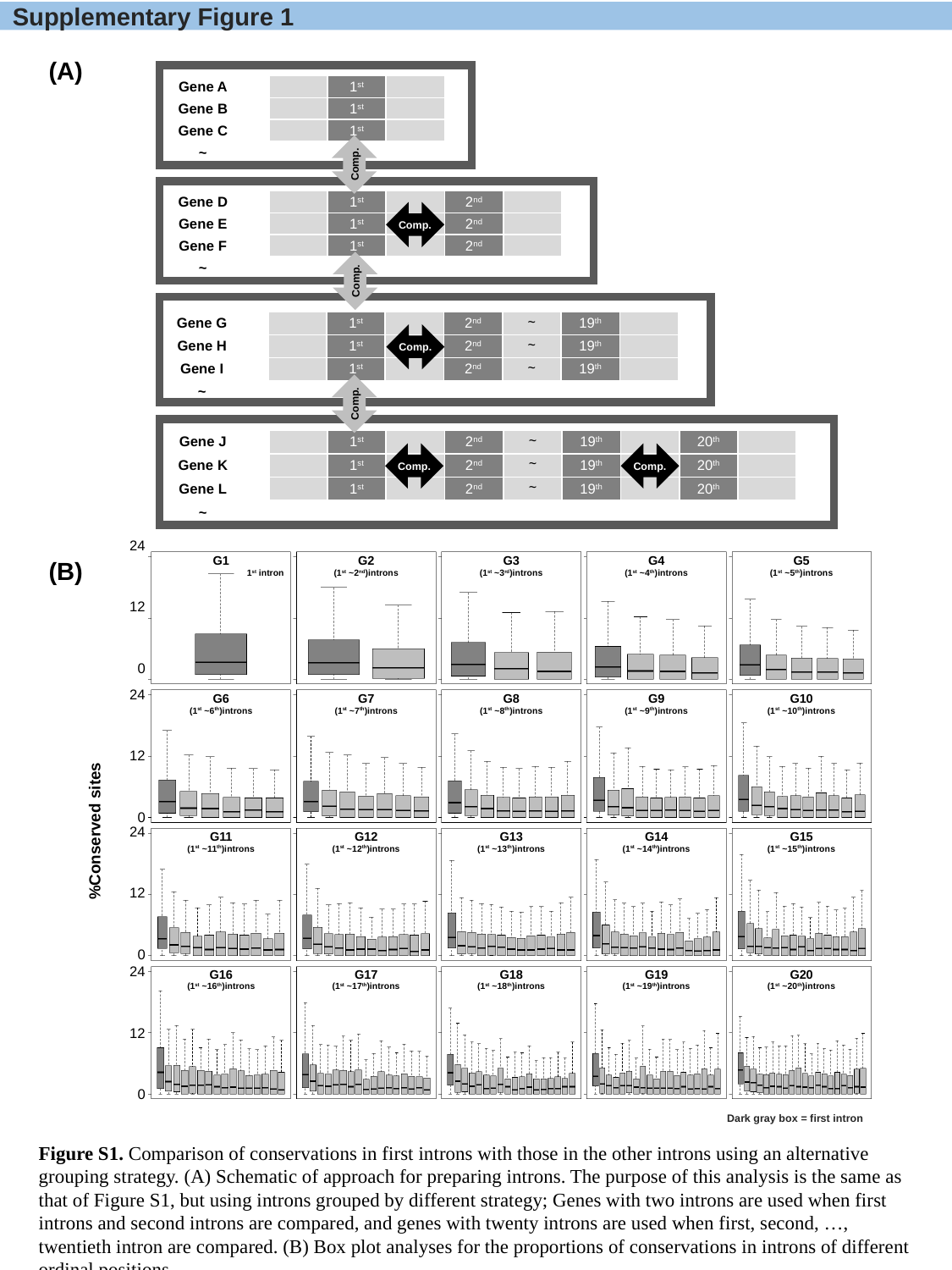

Supplementary Figure 1
(A)
| Gene A | | 1st | |
| --- | --- | --- | --- |
| Gene B | | 1st | |
| Gene C | | 1st | |
| ~ | | 1st | |
Comp.
| Gene D | | 1st | | 2nd | |
| --- | --- | --- | --- | --- | --- |
| Gene E | | 1st | | 2nd | |
| Gene F | | 1st | | 2nd | |
| ~ | | 1st | | 2nd | |
Comp.
Comp.
| Gene G | | 1st | | 2nd | ~ | 19th | |
| --- | --- | --- | --- | --- | --- | --- | --- |
| Gene H | | 1st | | 2nd | ~ | 19th | |
| Gene I | | 1st | | 2nd | ~ | 19th | |
| ~ | | 1st | | 2nd | | 19th | |
Comp.
Comp.
| Gene J | | 1st | | 2nd | ~ | 19th | | 20th | |
| --- | --- | --- | --- | --- | --- | --- | --- | --- | --- |
| Gene K | | 1st | | 2nd | ~ | 19th | | 20th | |
| Gene L | | 1st | | 2nd | ~ | 19th | | 20th | |
| ~ | | 1st | | 2nd | | 19th | | 20th | |
Comp.
Comp.
| 24 |
| --- |
| 12 |
| 0 |
(B)
| G1 1st intron | G2 (1st ~2nd)introns | G3 (1st ~3rd)introns | G4 (1st ~4th)introns | G5 (1st ~5th)introns |
| --- | --- | --- | --- | --- |
| G6 (1st ~6th)introns | G7 (1st ~7th)introns | G8 (1st ~8th)introns | G9 (1st ~9th)introns | G10 (1st ~10th)introns |
| G11 (1st ~11th)introns | G12 (1st ~12th)introns | G13 (1st ~13th)introns | G14 (1st ~14th)introns | G15 (1st ~15th)introns |
| G16 (1st ~16th)introns | G17 (1st ~17th)introns | G18 (1st ~18th)introns | G19 (1st ~19th)introns | G20 (1st ~20th)introns |
| 24 |
| --- |
| 12 |
| 0 |
| %Conserved sites |
| --- |
| 24 |
| --- |
| 12 |
| 0 |
| 24 |
| --- |
| 12 |
| 0 |
Dark gray box = first intron
Figure S1. Comparison of conservations in first introns with those in the other introns using an alternative grouping strategy. (A) Schematic of approach for preparing introns. The purpose of this analysis is the same as that of Figure S1, but using introns grouped by different strategy; Genes with two introns are used when first introns and second introns are compared, and genes with twenty introns are used when first, second, …, twentieth intron are compared. (B) Box plot analyses for the proportions of conservations in introns of different ordinal positions.

## Slide 2
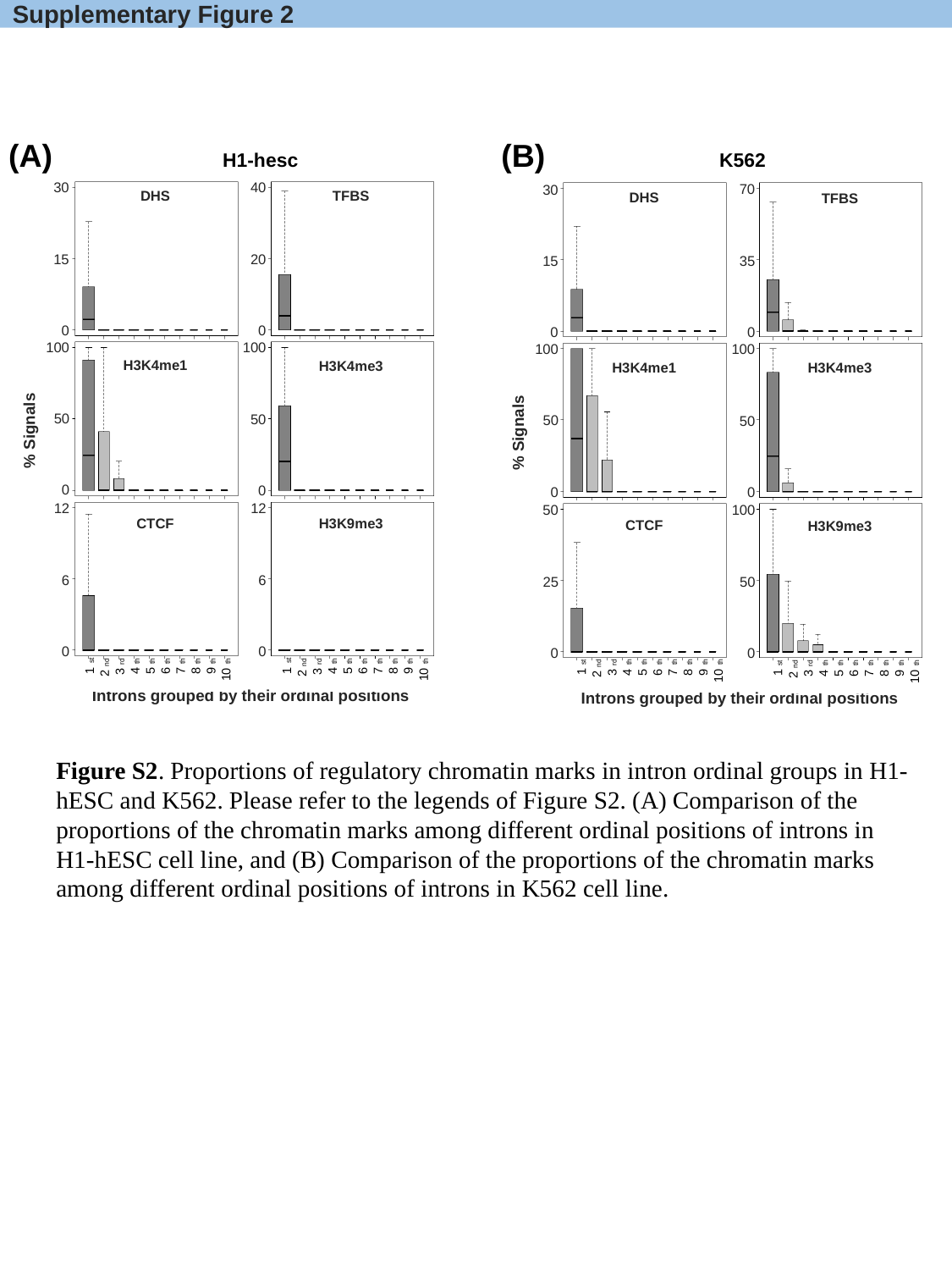

Supplementary Figure 2
(A) H1-hesc
(B) K562
DHS
40
TFBS
30
DHS
70
TFBS
30
20
15
35
15
0
0
0
0
100
100
100
100
H3K4me1
H3K4me3
H3K4me1
H3K4me3
50
50
50
50
% Signals
% Signals
0
0
0
0
12
12
50
100
CTCF
H3K9me3
CTCF
H3K9me3
6
6
25
50
0
0
0
0
| 1st | 2nd | 3rd | 4th | 5th | 6th | 7th | 8th | 9th | 10th |
| --- | --- | --- | --- | --- | --- | --- | --- | --- | --- |
| 1st | 2nd | 3rd | 4th | 5th | 6th | 7th | 8th | 9th | 10th |
| --- | --- | --- | --- | --- | --- | --- | --- | --- | --- |
| 1st | 2nd | 3rd | 4th | 5th | 6th | 7th | 8th | 9th | 10th |
| --- | --- | --- | --- | --- | --- | --- | --- | --- | --- |
| 1st | 2nd | 3rd | 4th | 5th | 6th | 7th | 8th | 9th | 10th |
| --- | --- | --- | --- | --- | --- | --- | --- | --- | --- |
Introns grouped by their ordinal positions
Introns grouped by their ordinal positions
Figure S2. Proportions of regulatory chromatin marks in intron ordinal groups in H1-hESC and K562. Please refer to the legends of Figure S2. (A) Comparison of the proportions of the chromatin marks among different ordinal positions of introns in H1-hESC cell line, and (B) Comparison of the proportions of the chromatin marks among different ordinal positions of introns in K562 cell line.

## Slide 3
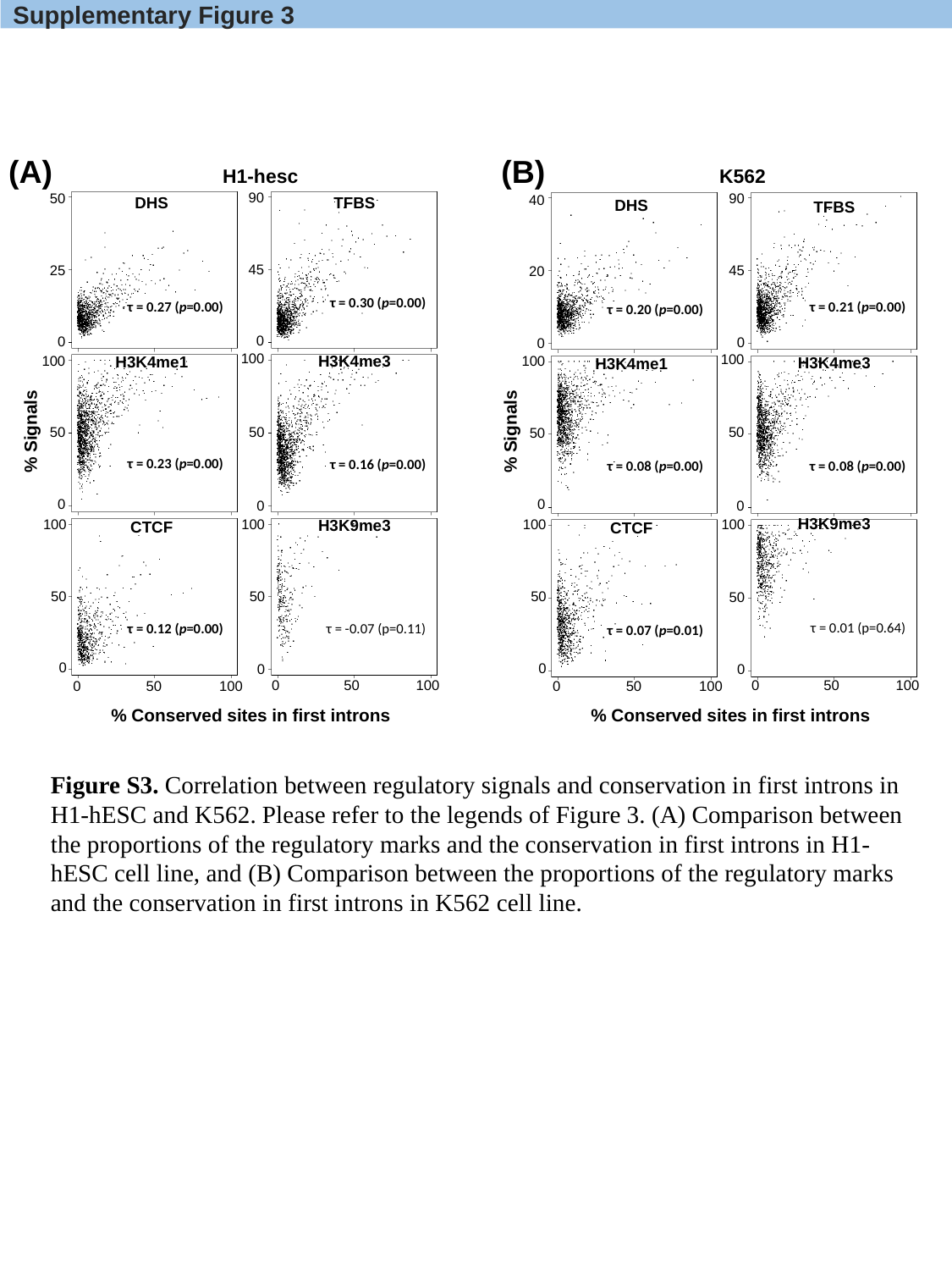

Supplementary Figure 3
(A) H1-hesc
(B) K562
| 90 |
| --- |
| 45 |
| 0 |
| 50 |
| --- |
| 25 |
| 0 |
| 90 |
| --- |
| 45 |
| 0 |
| 40 |
| --- |
| 20 |
| 0 |
| DHS τ = 0.27 (p=0.00) |
| --- |
| H3K4me1 τ = 0.23 (p=0.00) |
| CTCF τ = 0.12 (p=0.00) |
| TFBS τ = 0.30 (p=0.00) |
| --- |
| H3K4me3 τ = 0.16 (p=0.00) |
| H3K9me3 τ = -0.07 (p=0.11) |
| DHS τ = 0.20 (p=0.00) |
| --- |
| H3K4me1 τ = 0.08 (p=0.00) |
| CTCF τ = 0.07 (p=0.01) |
| TFBS τ = 0.21 (p=0.00) |
| --- |
| H3K4me3 τ = 0.08 (p=0.00) |
| H3K9me3 τ = 0.01 (p=0.64) |
| % Signals |
| --- |
| % Signals |
| --- |
| 100 |
| --- |
| 50 |
| 0 |
| 100 |
| --- |
| 50 |
| 0 |
| 100 |
| --- |
| 50 |
| 0 |
| 100 |
| --- |
| 50 |
| 0 |
| 100 |
| --- |
| 50 |
| 0 |
| 100 |
| --- |
| 50 |
| 0 |
| 100 |
| --- |
| 50 |
| 0 |
| 100 |
| --- |
| 50 |
| 0 |
| 0 | 50 | 100 |
| --- | --- | --- |
| 0 | 50 | 100 |
| --- | --- | --- |
| 0 | 50 | 100 |
| --- | --- | --- |
| 0 | 50 | 100 |
| --- | --- | --- |
| % Conserved sites in first introns |
| --- |
| % Conserved sites in first introns |
| --- |
Figure S3. Correlation between regulatory signals and conservation in first introns in H1-hESC and K562. Please refer to the legends of Figure 3. (A) Comparison between the proportions of the regulatory marks and the conservation in first introns in H1-hESC cell line, and (B) Comparison between the proportions of the regulatory marks and the conservation in first introns in K562 cell line.

## Slide 4
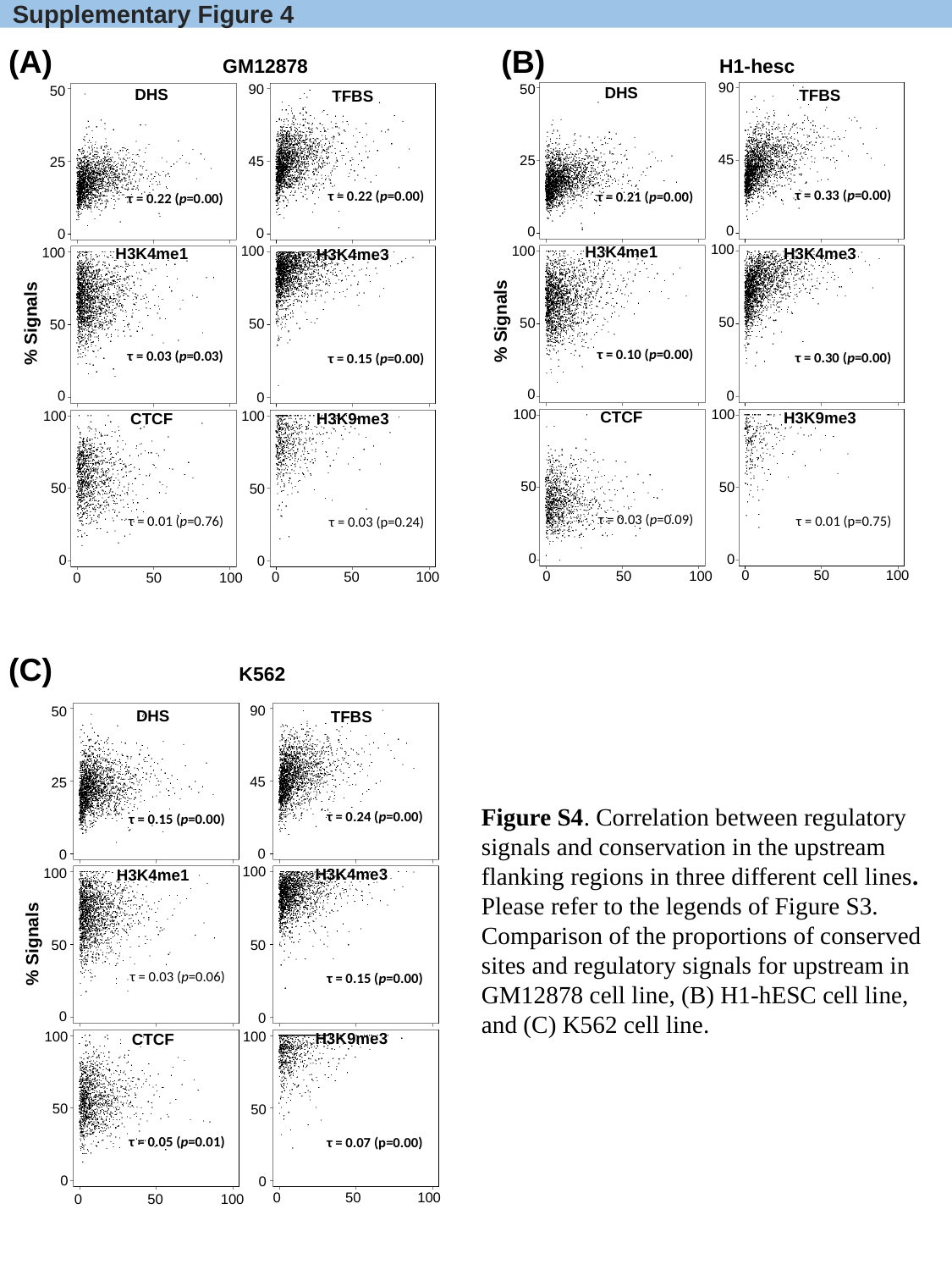

Supplementary Figure 4
(A) GM12878
(B) H1-hesc
| 90 |
| --- |
| 45 |
| 0 |
| 50 |
| --- |
| 25 |
| 0 |
| 90 |
| --- |
| 45 |
| 0 |
| 50 |
| --- |
| 25 |
| 0 |
| DHS τ = 0.21 (p=0.00) |
| --- |
| H3K4me1 τ = 0.10 (p=0.00) |
| CTCF τ = 0.03 (p=0.09) |
| DHS τ = 0.22 (p=0.00) |
| --- |
| H3K4me1 τ = 0.03 (p=0.03) |
| CTCF τ = 0.01 (p=0.76) |
| TFBS τ = 0.33 (p=0.00) |
| --- |
| H3K4me3 τ = 0.30 (p=0.00) |
| H3K9me3 τ = 0.01 (p=0.75) |
| TFBS τ = 0.22 (p=0.00) |
| --- |
| H3K4me3 τ = 0.15 (p=0.00) |
| H3K9me3 τ = 0.03 (p=0.24) |
| % Signals |
| --- |
| % Signals |
| --- |
| 100 |
| --- |
| 50 |
| 0 |
| 100 |
| --- |
| 50 |
| 0 |
| 100 |
| --- |
| 50 |
| 0 |
| 100 |
| --- |
| 50 |
| 0 |
| 100 |
| --- |
| 50 |
| 0 |
| 100 |
| --- |
| 50 |
| 0 |
| 100 |
| --- |
| 50 |
| 0 |
| 100 |
| --- |
| 50 |
| 0 |
| 0 | 50 | 100 |
| --- | --- | --- |
| 0 | 50 | 100 |
| --- | --- | --- |
| 0 | 50 | 100 |
| --- | --- | --- |
| 0 | 50 | 100 |
| --- | --- | --- |
(C) K562
| 90 |
| --- |
| 45 |
| 0 |
| 50 |
| --- |
| 25 |
| 0 |
| DHS τ = 0.15 (p=0.00) |
| --- |
| H3K4me1 τ = 0.03 (p=0.06) |
| CTCF τ = 0.05 (p=0.01) |
| TFBS τ = 0.24 (p=0.00) |
| --- |
| H3K4me3 τ = 0.15 (p=0.00) |
| H3K9me3 τ = 0.07 (p=0.00) |
Figure S4. Correlation between regulatory signals and conservation in the upstream flanking regions in three different cell lines. Please refer to the legends of Figure S3. Comparison of the proportions of conserved sites and regulatory signals for upstream in GM12878 cell line, (B) H1-hESC cell line, and (C) K562 cell line.
| % Signals |
| --- |
| 100 |
| --- |
| 50 |
| 0 |
| 100 |
| --- |
| 50 |
| 0 |
| 100 |
| --- |
| 50 |
| 0 |
| 100 |
| --- |
| 50 |
| 0 |
| 0 | 50 | 100 |
| --- | --- | --- |
| 0 | 50 | 100 |
| --- | --- | --- |

## Slide 5
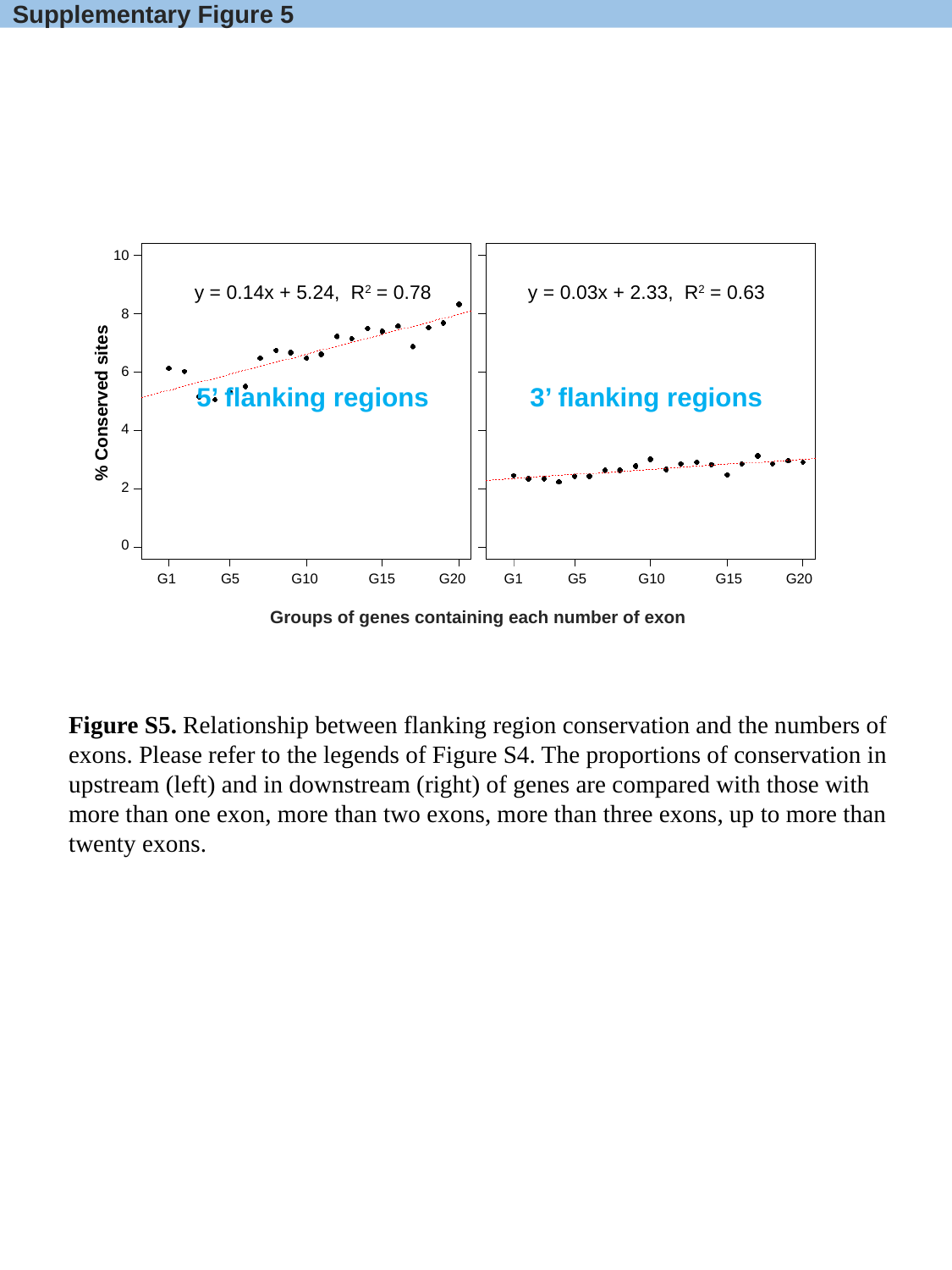

Supplementary Figure 5
| 10 |
| --- |
| 8 |
| 6 |
| 4 |
| 2 |
| 0 |
| y = 0.14x + 5.24, R2 = 0.78 5’ flanking regions | y = 0.03x + 2.33, R2 = 0.63 3’ flanking regions |
| --- | --- |
| % Conserved sites |
| --- |
| G1 | G5 | G10 | G15 | G20 |
| --- | --- | --- | --- | --- |
| G1 | G5 | G10 | G15 | G20 |
| --- | --- | --- | --- | --- |
Groups of genes containing each number of exon
Figure S5. Relationship between flanking region conservation and the numbers of exons. Please refer to the legends of Figure S4. The proportions of conservation in upstream (left) and in downstream (right) of genes are compared with those with more than one exon, more than two exons, more than three exons, up to more than twenty exons.

## Slide 6
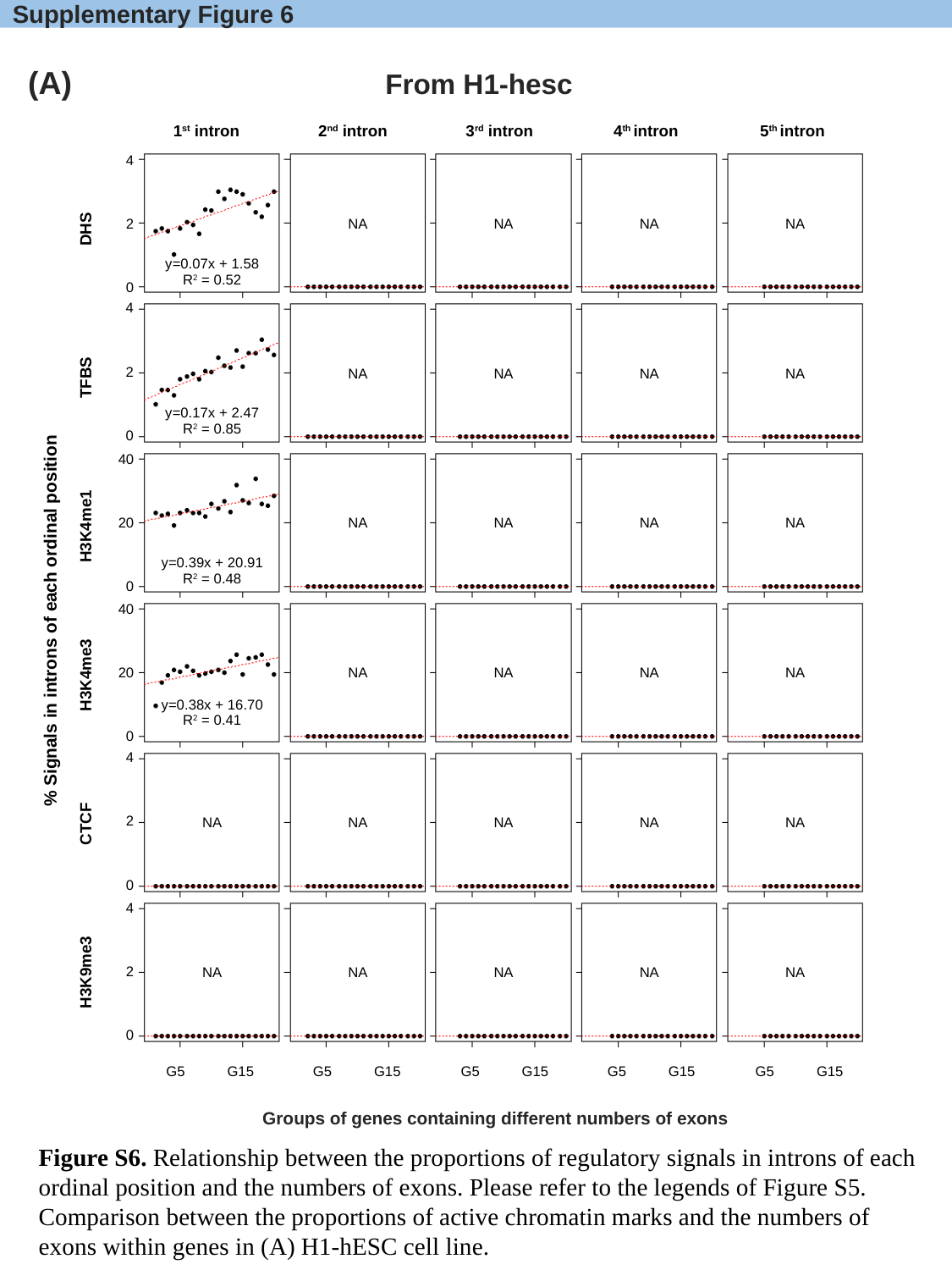

Supplementary Figure 6
 (A) From H1-hesc
| 1st intron | 2nd intron | 3rd intron | 4th intron | 5th intron |
| --- | --- | --- | --- | --- |
| y=0.07x + 1.58 R2 = 0.52 | NA | NA | NA | NA |
| --- | --- | --- | --- | --- |
| y=0.17x + 2.47 R2 = 0.85 | NA | NA | NA | NA |
| y=0.39x + 20.91 R2 = 0.48 | NA | NA | NA | NA |
| y=0.38x + 16.70 R2 = 0.41 | NA | NA | NA | NA |
| NA | NA | NA | NA | NA |
| NA | NA | NA | NA | NA |
| 4 |
| --- |
| 2 |
| 0 |
| DHS |
| --- |
| TFBS |
| H3K4me1 |
| H3K4me3 |
| CTCF |
| H3K9me3 |
| 4 |
| --- |
| 2 |
| 0 |
| % Signals in introns of each ordinal position |
| --- |
| 40 |
| --- |
| 20 |
| 0 |
| 40 |
| --- |
| 20 |
| 0 |
| 4 |
| --- |
| 2 |
| 0 |
| 4 |
| --- |
| 2 |
| 0 |
| G5 | G15 |
| --- | --- |
| G5 | G15 |
| --- | --- |
| G5 | G15 |
| --- | --- |
| G5 | G15 |
| --- | --- |
| G5 | G15 |
| --- | --- |
Groups of genes containing different numbers of exons
Figure S6. Relationship between the proportions of regulatory signals in introns of each ordinal position and the numbers of exons. Please refer to the legends of Figure S5. Comparison between the proportions of active chromatin marks and the numbers of exons within genes in (A) H1-hESC cell line.

## Slide 7
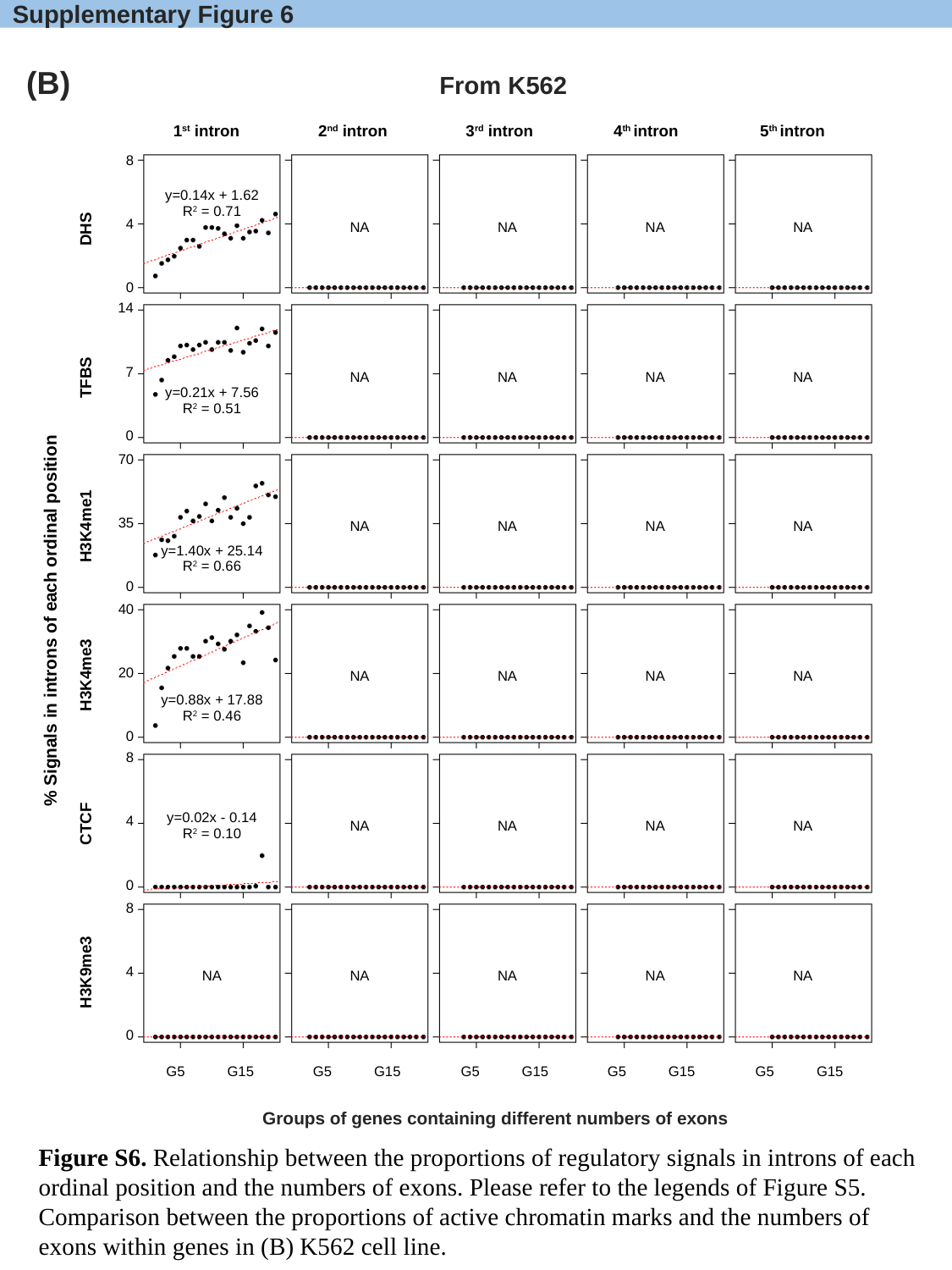

Supplementary Figure 6
 (B) From K562
| 1st intron | 2nd intron | 3rd intron | 4th intron | 5th intron |
| --- | --- | --- | --- | --- |
| y=0.14x + 1.62 R2 = 0.71 | NA | NA | NA | NA |
| --- | --- | --- | --- | --- |
| y=0.21x + 7.56 R2 = 0.51 | NA | NA | NA | NA |
| y=1.40x + 25.14 R2 = 0.66 | NA | NA | NA | NA |
| y=0.88x + 17.88 R2 = 0.46 | NA | NA | NA | NA |
| y=0.02x - 0.14 R2 = 0.10 | NA | NA | NA | NA |
| NA | NA | NA | NA | NA |
| 8 |
| --- |
| 4 |
| 0 |
| DHS |
| --- |
| TFBS |
| H3K4me1 |
| H3K4me3 |
| CTCF |
| H3K9me3 |
| 14 |
| --- |
| 7 |
| 0 |
| % Signals in introns of each ordinal position |
| --- |
| 70 |
| --- |
| 35 |
| 0 |
| 40 |
| --- |
| 20 |
| 0 |
| 8 |
| --- |
| 4 |
| 0 |
| 8 |
| --- |
| 4 |
| 0 |
| G5 | G15 |
| --- | --- |
| G5 | G15 |
| --- | --- |
| G5 | G15 |
| --- | --- |
| G5 | G15 |
| --- | --- |
| G5 | G15 |
| --- | --- |
Groups of genes containing different numbers of exons
Figure S6. Relationship between the proportions of regulatory signals in introns of each ordinal position and the numbers of exons. Please refer to the legends of Figure S5. Comparison between the proportions of active chromatin marks and the numbers of exons within genes in (B) K562 cell line.

## Slide 8
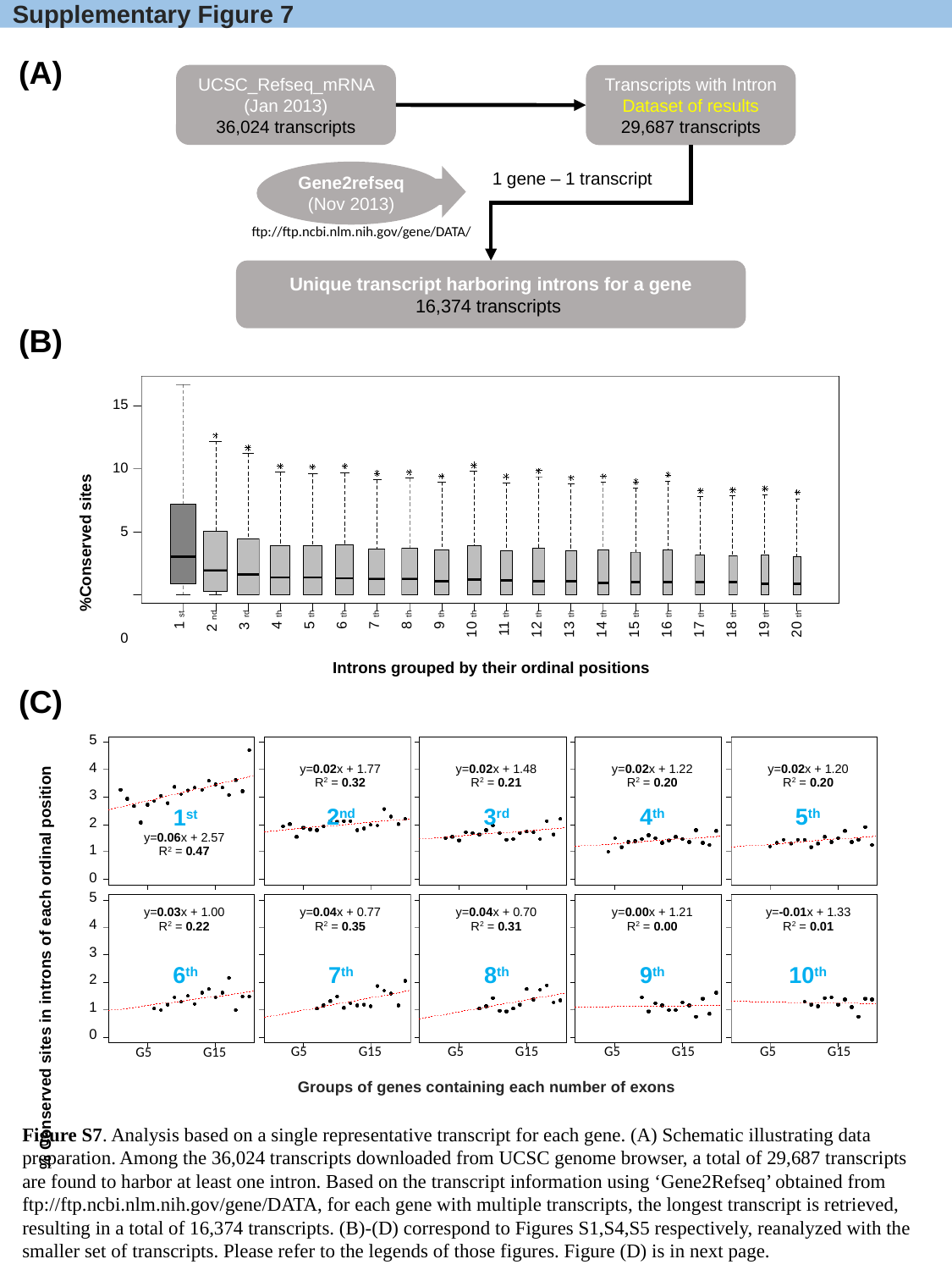

Supplementary Figure 7
(A)
UCSC_Refseq_mRNA (Jan 2013)
36,024 transcripts
Transcripts with Intron
Dataset of results
29,687 transcripts
1 gene – 1 transcript
Gene2refseq
(Nov 2013)
ftp://ftp.ncbi.nlm.nih.gov/gene/DATA/
Unique transcript harboring introns for a gene
16,374 transcripts
(B)
| %Conserved sites | 15 |
| --- | --- |
| | 10 |
| | 5 |
| | 0 |
| 1st | 2nd | 3rd | 4th | 5th | 6th | 7th | 8th | 9th | 10th | 11th | 12th | 13th | 14th | 15th | 16th | 17th | 18th | 19th | 20th |
| --- | --- | --- | --- | --- | --- | --- | --- | --- | --- | --- | --- | --- | --- | --- | --- | --- | --- | --- | --- |
| Introns grouped by their ordinal positions | | | | | | | | | | | | | | | | | | | |
(C)
| 5 |
| --- |
| 4 |
| 3 |
| 2 |
| 1 |
| 0 |
| 1st | 2nd | 3rd | 4th | 5th |
| --- | --- | --- | --- | --- |
| 6th | 7th | 8th | 9th | 10th |
| y=0.06x + 2.57 R2 = 0.47 | y=0.02x + 1.77 R2 = 0.32 | y=0.02x + 1.48 R2 = 0.21 | y=0.02x + 1.22 R2 = 0.20 | y=0.02x + 1.20 R2 = 0.20 |
| --- | --- | --- | --- | --- |
| y=0.03x + 1.00 R2 = 0.22 | y=0.04x + 0.77 R2 = 0.35 | y=0.04x + 0.70 R2 = 0.31 | y=0.00x + 1.21 R2 = 0.00 | y=-0.01x + 1.33 R2 = 0.01 |
| % Conserved sites in introns of each ordinal position |
| --- |
| 5 |
| --- |
| 4 |
| 3 |
| 2 |
| 1 |
| 0 |
| G5 | G15 |
| --- | --- |
| G5 | G15 |
| --- | --- |
| G5 | G15 |
| --- | --- |
| G5 | G15 |
| --- | --- |
| G5 | G15 |
| --- | --- |
Groups of genes containing each number of exons
Figure S7. Analysis based on a single representative transcript for each gene. (A) Schematic illustrating data preparation. Among the 36,024 transcripts downloaded from UCSC genome browser, a total of 29,687 transcripts are found to harbor at least one intron. Based on the transcript information using ‘Gene2Refseq’ obtained from ftp://ftp.ncbi.nlm.nih.gov/gene/DATA, for each gene with multiple transcripts, the longest transcript is retrieved, resulting in a total of 16,374 transcripts. (B)-(D) correspond to Figures S1,S4,S5 respectively, reanalyzed with the smaller set of transcripts. Please refer to the legends of those figures. Figure (D) is in next page.

## Slide 9
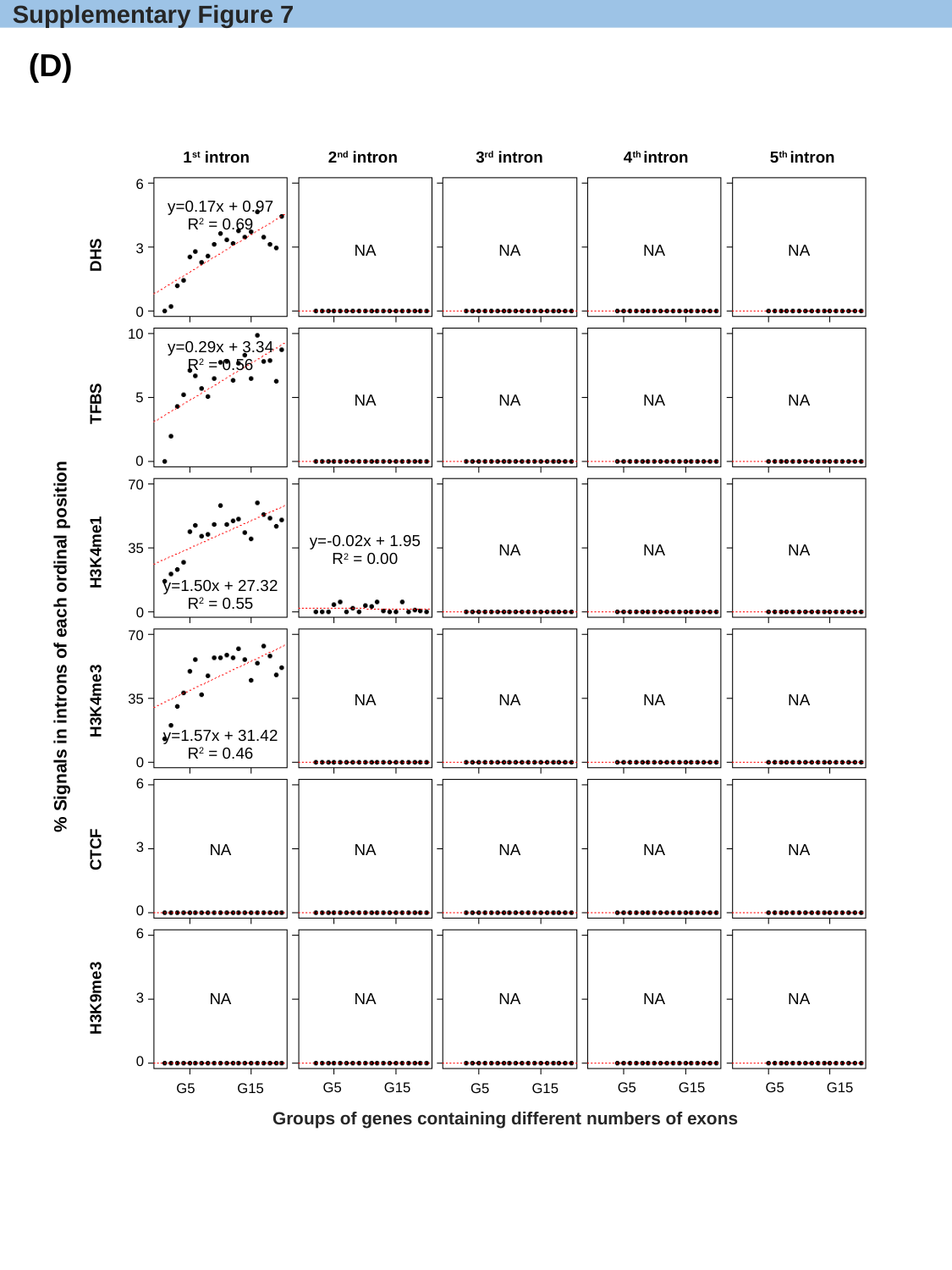

Supplementary Figure 7
(D)
| 1st intron | 2nd intron | 3rd intron | 4th intron | 5th intron |
| --- | --- | --- | --- | --- |
| y=0.17x + 0.97 R2 = 0.69 | NA | NA | NA | NA |
| --- | --- | --- | --- | --- |
| y=0.29x + 3.34 R2 = 0.56 | NA | NA | NA | NA |
| y=1.50x + 27.32 R2 = 0.55 | y=-0.02x + 1.95 R2 = 0.00 | NA | NA | NA |
| y=1.57x + 31.42 R2 = 0.46 | NA | NA | NA | NA |
| NA | NA | NA | NA | NA |
| NA | NA | NA | NA | NA |
| 6 |
| --- |
| 3 |
| 0 |
| DHS |
| --- |
| TFBS |
| H3K4me1 |
| H3K4me3 |
| CTCF |
| H3K9me3 |
| 10 |
| --- |
| 5 |
| 0 |
| % Signals in introns of each ordinal position |
| --- |
| 70 |
| --- |
| 35 |
| 0 |
| 70 |
| --- |
| 35 |
| 0 |
| 6 |
| --- |
| 3 |
| 0 |
| 6 |
| --- |
| 3 |
| 0 |
| G5 | G15 |
| --- | --- |
| G5 | G15 |
| --- | --- |
| G5 | G15 |
| --- | --- |
| G5 | G15 |
| --- | --- |
| G5 | G15 |
| --- | --- |
Groups of genes containing different numbers of exons

## Slide 10
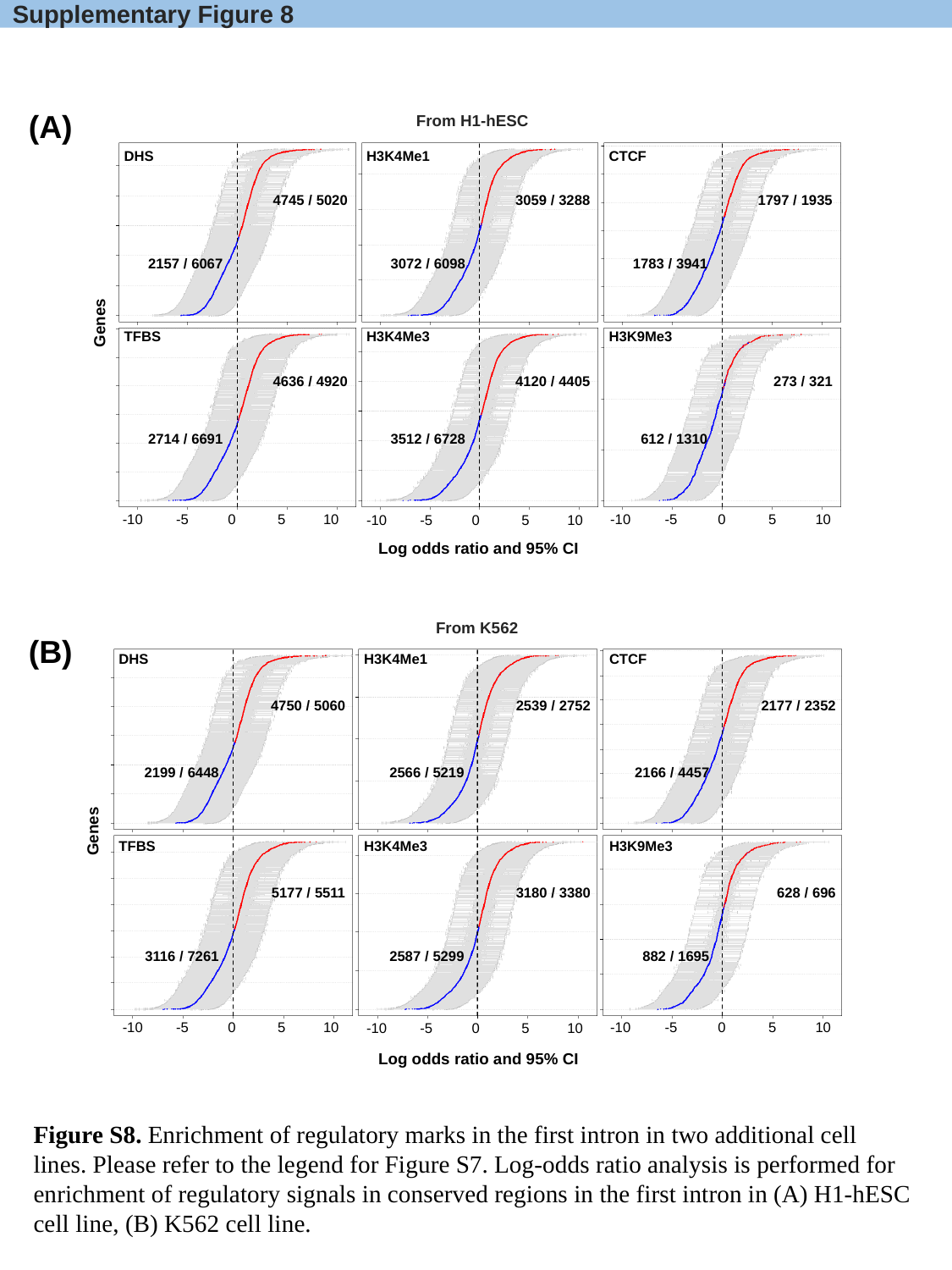

Supplementary Figure 8
(A)
From H1-hESC
| DHS | 4745 / 5020 | H3K4Me1 | 3059 / 3288 | CTCF | 1797 / 1935 |
| --- | --- | --- | --- | --- | --- |
| 2157 / 6067 | | 3072 / 6098 | | 1783 / 3941 | |
| TFBS | 4636 / 4920 | H3K4Me3 | 4120 / 4405 | H3K9Me3 | 273 / 321 |
| 2714 / 6691 | | 3512 / 6728 | | 612 / 1310 | |
| Genes |
| --- |
| -10 | -5 | 0 | 5 | 10 |
| --- | --- | --- | --- | --- |
| -10 | -5 | 0 | 5 | 10 |
| --- | --- | --- | --- | --- |
| -10 | -5 | 0 | 5 | 10 |
| --- | --- | --- | --- | --- |
| Log odds ratio and 95% CI |
| --- |
From K562
(B)
| DHS | 4750 / 5060 | H3K4Me1 | 2539 / 2752 | CTCF | 2177 / 2352 |
| --- | --- | --- | --- | --- | --- |
| 2199 / 6448 | | 2566 / 5219 | | 2166 / 4457 | |
| TFBS | 5177 / 5511 | H3K4Me3 | 3180 / 3380 | H3K9Me3 | 628 / 696 |
| 3116 / 7261 | | 2587 / 5299 | | 882 / 1695 | |
| Genes |
| --- |
| -10 | -5 | 0 | 5 | 10 |
| --- | --- | --- | --- | --- |
| -10 | -5 | 0 | 5 | 10 |
| --- | --- | --- | --- | --- |
| -10 | -5 | 0 | 5 | 10 |
| --- | --- | --- | --- | --- |
| Log odds ratio and 95% CI |
| --- |
Figure S8. Enrichment of regulatory marks in the first intron in two additional cell lines. Please refer to the legend for Figure S7. Log-odds ratio analysis is performed for enrichment of regulatory signals in conserved regions in the first intron in (A) H1-hESC cell line, (B) K562 cell line.

## Slide 11
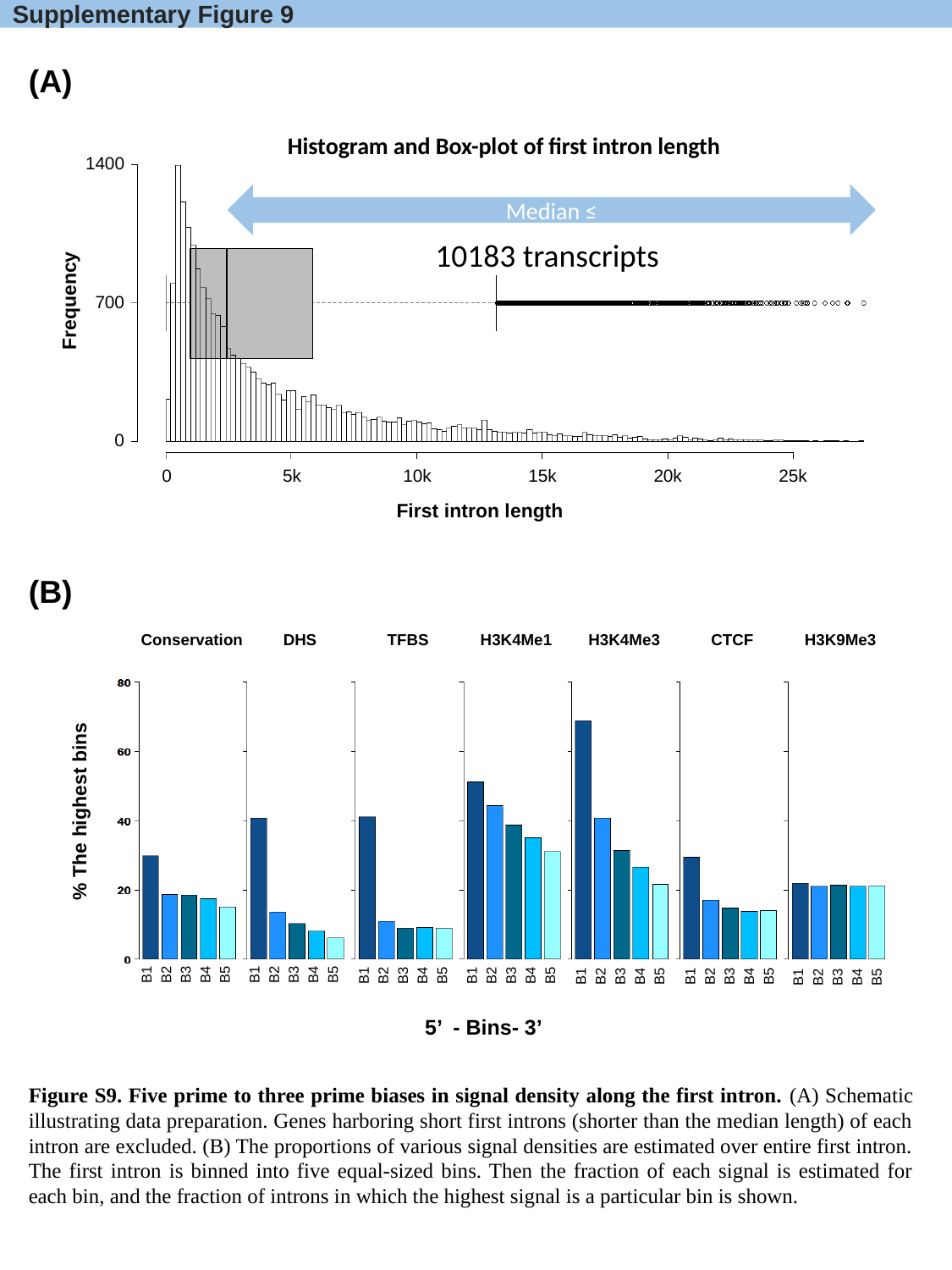

Supplementary Figure 9
(A)
| 1400 |
| --- |
| 700 |
| 0 |
Histogram and Box-plot of first intron length
| Frequency |
| --- |
Median ≤
10183 transcripts
| 0 | 5k | 10k | 15k | 20k | 25k |
| --- | --- | --- | --- | --- | --- |
| First intron length | | | | | |
(B)
| Conservation | DHS | TFBS | H3K4Me1 | H3K4Me3 | CTCF | H3K9Me3 |
| --- | --- | --- | --- | --- | --- | --- |
| % The highest bins |
| --- |
| B1 | B2 | B3 | B4 | B5 |
| --- | --- | --- | --- | --- |
| B1 | B2 | B3 | B4 | B5 |
| --- | --- | --- | --- | --- |
| B1 | B2 | B3 | B4 | B5 |
| --- | --- | --- | --- | --- |
| B1 | B2 | B3 | B4 | B5 |
| --- | --- | --- | --- | --- |
| B1 | B2 | B3 | B4 | B5 |
| --- | --- | --- | --- | --- |
| B1 | B2 | B3 | B4 | B5 |
| --- | --- | --- | --- | --- |
| B1 | B2 | B3 | B4 | B5 |
| --- | --- | --- | --- | --- |
5’ - Bins- 3’
Figure S9. Five prime to three prime biases in signal density along the first intron. (A) Schematic illustrating data preparation. Genes harboring short first introns (shorter than the median length) of each intron are excluded. (B) The proportions of various signal densities are estimated over entire first intron. The first intron is binned into five equal-sized bins. Then the fraction of each signal is estimated for each bin, and the fraction of introns in which the highest signal is a particular bin is shown.

## Slide 12
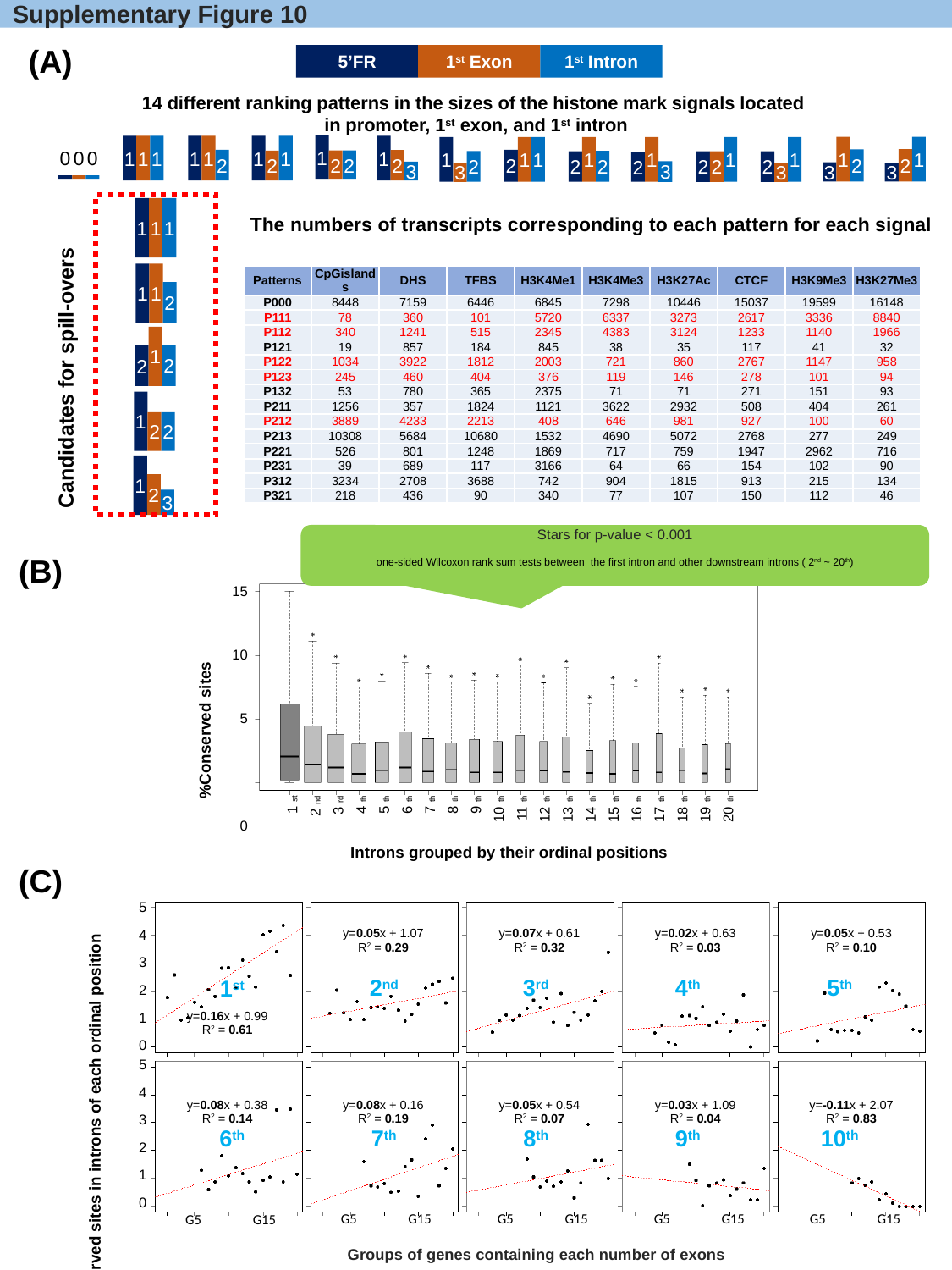

Supplementary Figure 10
(A)
5’FR
1st Exon
1st Intron
14 different ranking patterns in the sizes of the histone mark signals located
in promoter, 1st exon, and 1st intron
0
0
0
1
2
2
1
1
1
1
2
3
1
1
2
1
1
2
1
2
3
1
2
3
1
2
3
1
1
2
1
2
2
1
2
2
1
2
3
1
2
3
1
1
1
The numbers of transcripts corresponding to each pattern for each signal
1
1
2
| Patterns | CpGislands | DHS | TFBS | H3K4Me1 | H3K4Me3 | H3K27Ac | CTCF | H3K9Me3 | H3K27Me3 |
| --- | --- | --- | --- | --- | --- | --- | --- | --- | --- |
| P000 | 8448 | 7159 | 6446 | 6845 | 7298 | 10446 | 15037 | 19599 | 16148 |
| P111 | 78 | 360 | 101 | 5720 | 6337 | 3273 | 2617 | 3336 | 8840 |
| P112 | 340 | 1241 | 515 | 2345 | 4383 | 3124 | 1233 | 1140 | 1966 |
| P121 | 19 | 857 | 184 | 845 | 38 | 35 | 117 | 41 | 32 |
| P122 | 1034 | 3922 | 1812 | 2003 | 721 | 860 | 2767 | 1147 | 958 |
| P123 | 245 | 460 | 404 | 376 | 119 | 146 | 278 | 101 | 94 |
| P132 | 53 | 780 | 365 | 2375 | 71 | 71 | 271 | 151 | 93 |
| P211 | 1256 | 357 | 1824 | 1121 | 3622 | 2932 | 508 | 404 | 261 |
| P212 | 3889 | 4233 | 2213 | 408 | 646 | 981 | 927 | 100 | 60 |
| P213 | 10308 | 5684 | 10680 | 1532 | 4690 | 5072 | 2768 | 277 | 249 |
| P221 | 526 | 801 | 1248 | 1869 | 717 | 759 | 1947 | 2962 | 716 |
| P231 | 39 | 689 | 117 | 3166 | 64 | 66 | 154 | 102 | 90 |
| P312 | 3234 | 2708 | 3688 | 742 | 904 | 1815 | 913 | 215 | 134 |
| P321 | 218 | 436 | 90 | 340 | 77 | 107 | 150 | 112 | 46 |
1
2
2
Candidates for spill-overs
1
2
2
1
2
3
Stars for p-value < 0.001
one-sided Wilcoxon rank sum tests between the first intron and other downstream introns ( 2nd ~ 20th)
(B)
| %Conserved sites | 15 |
| --- | --- |
| | 10 |
| | 5 |
| | 0 |
| 1st | 2nd | 3rd | 4th | 5th | 6th | 7th | 8th | 9th | 10th | 11th | 12th | 13th | 14th | 15th | 16th | 17th | 18th | 19th | 20th |
| --- | --- | --- | --- | --- | --- | --- | --- | --- | --- | --- | --- | --- | --- | --- | --- | --- | --- | --- | --- |
| Introns grouped by their ordinal positions | | | | | | | | | | | | | | | | | | | |
(C)
| 5 |
| --- |
| 4 |
| 3 |
| 2 |
| 1 |
| 0 |
| y=0.16x + 0.99 R2 = 0.61 | y=0.05x + 1.07 R2 = 0.29 | y=0.07x + 0.61 R2 = 0.32 | y=0.02x + 0.63 R2 = 0.03 | y=0.05x + 0.53 R2 = 0.10 |
| --- | --- | --- | --- | --- |
| y=0.08x + 0.38 R2 = 0.14 | y=0.08x + 0.16 R2 = 0.19 | y=0.05x + 0.54 R2 = 0.07 | y=0.03x + 1.09 R2 = 0.04 | y=-0.11x + 2.07 R2 = 0.83 |
| 1st | 2nd | 3rd | 4th | 5th |
| --- | --- | --- | --- | --- |
| 6th | 7th | 8th | 9th | 10th |
| % Conserved sites in introns of each ordinal position |
| --- |
| 5 |
| --- |
| 4 |
| 3 |
| 2 |
| 1 |
| 0 |
| G5 | G15 |
| --- | --- |
| G5 | G15 |
| --- | --- |
| G5 | G15 |
| --- | --- |
| G5 | G15 |
| --- | --- |
| G5 | G15 |
| --- | --- |
Groups of genes containing each number of exons

## Slide 13
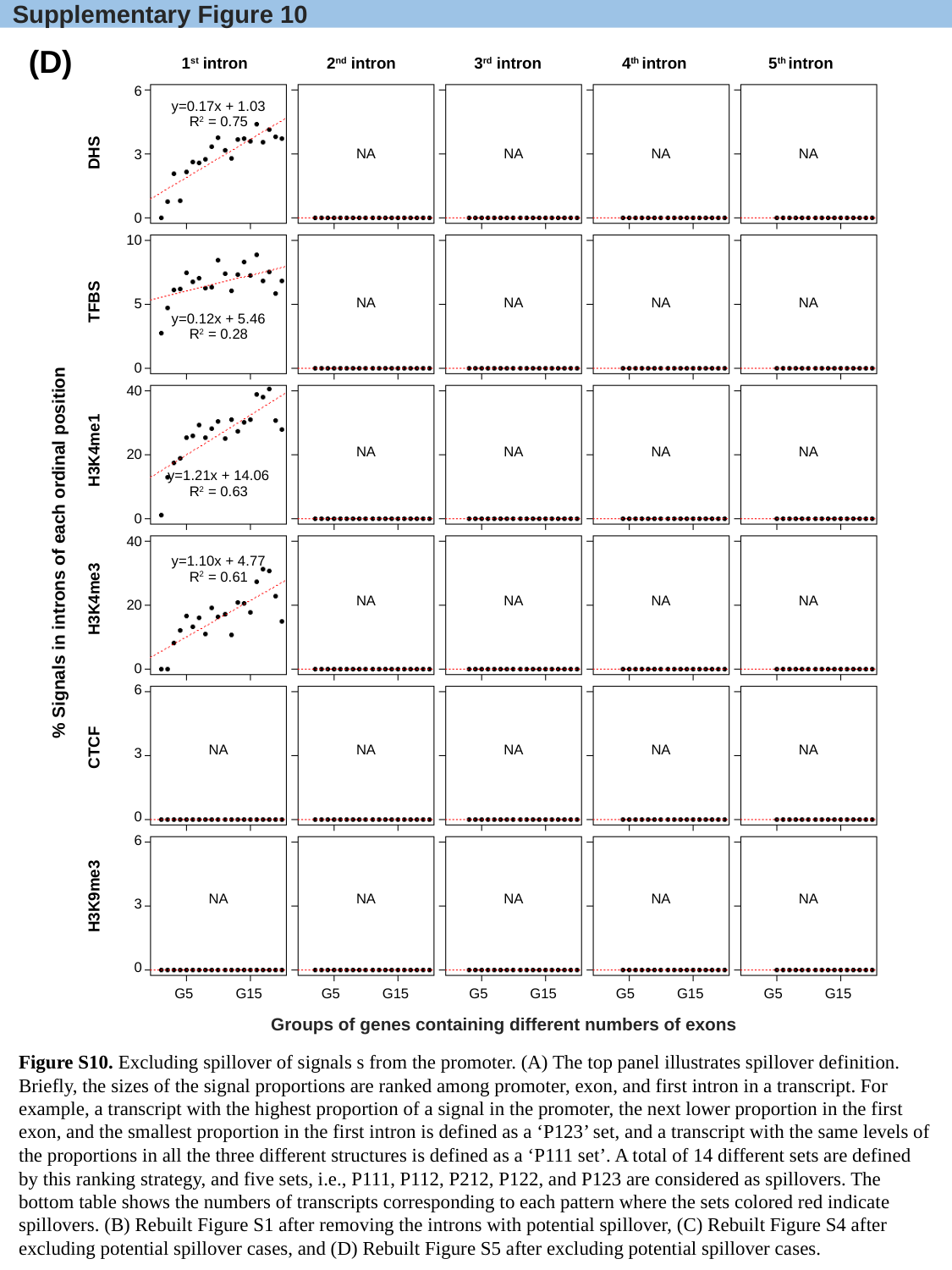

Supplementary Figure 10
(D)
| 1st intron | 2nd intron | 3rd intron | 4th intron | 5th intron |
| --- | --- | --- | --- | --- |
| DHS |
| --- |
| TFBS |
| H3K4me1 |
| H3K4me3 |
| CTCF |
| H3K9me3 |
| y=0.17x + 1.03 R2 = 0.75 | NA | NA | NA | NA |
| --- | --- | --- | --- | --- |
| y=0.12x + 5.46 R2 = 0.28 | NA | NA | NA | NA |
| y=1.21x + 14.06 R2 = 0.63 | NA | NA | NA | NA |
| y=1.10x + 4.77 R2 = 0.61 | NA | NA | NA | NA |
| NA | NA | NA | NA | NA |
| NA | NA | NA | NA | NA |
| 6 |
| --- |
| 3 |
| 0 |
| 10 |
| --- |
| 5 |
| 0 |
| % Signals in introns of each ordinal position |
| --- |
| 40 |
| --- |
| 20 |
| 0 |
| 40 |
| --- |
| 20 |
| 0 |
| 6 |
| --- |
| 3 |
| 0 |
| 6 |
| --- |
| 3 |
| 0 |
| G5 | G15 |
| --- | --- |
| G5 | G15 |
| --- | --- |
| G5 | G15 |
| --- | --- |
| G5 | G15 |
| --- | --- |
| G5 | G15 |
| --- | --- |
Groups of genes containing different numbers of exons
Figure S10. Excluding spillover of signals s from the promoter. (A) The top panel illustrates spillover definition. Briefly, the sizes of the signal proportions are ranked among promoter, exon, and first intron in a transcript. For example, a transcript with the highest proportion of a signal in the promoter, the next lower proportion in the first exon, and the smallest proportion in the first intron is defined as a ‘P123’ set, and a transcript with the same levels of the proportions in all the three different structures is defined as a ‘P111 set’. A total of 14 different sets are defined by this ranking strategy, and five sets, i.e., P111, P112, P212, P122, and P123 are considered as spillovers. The bottom table shows the numbers of transcripts corresponding to each pattern where the sets colored red indicate spillovers. (B) Rebuilt Figure S1 after removing the introns with potential spillover, (C) Rebuilt Figure S4 after excluding potential spillover cases, and (D) Rebuilt Figure S5 after excluding potential spillover cases.

## Slide 14
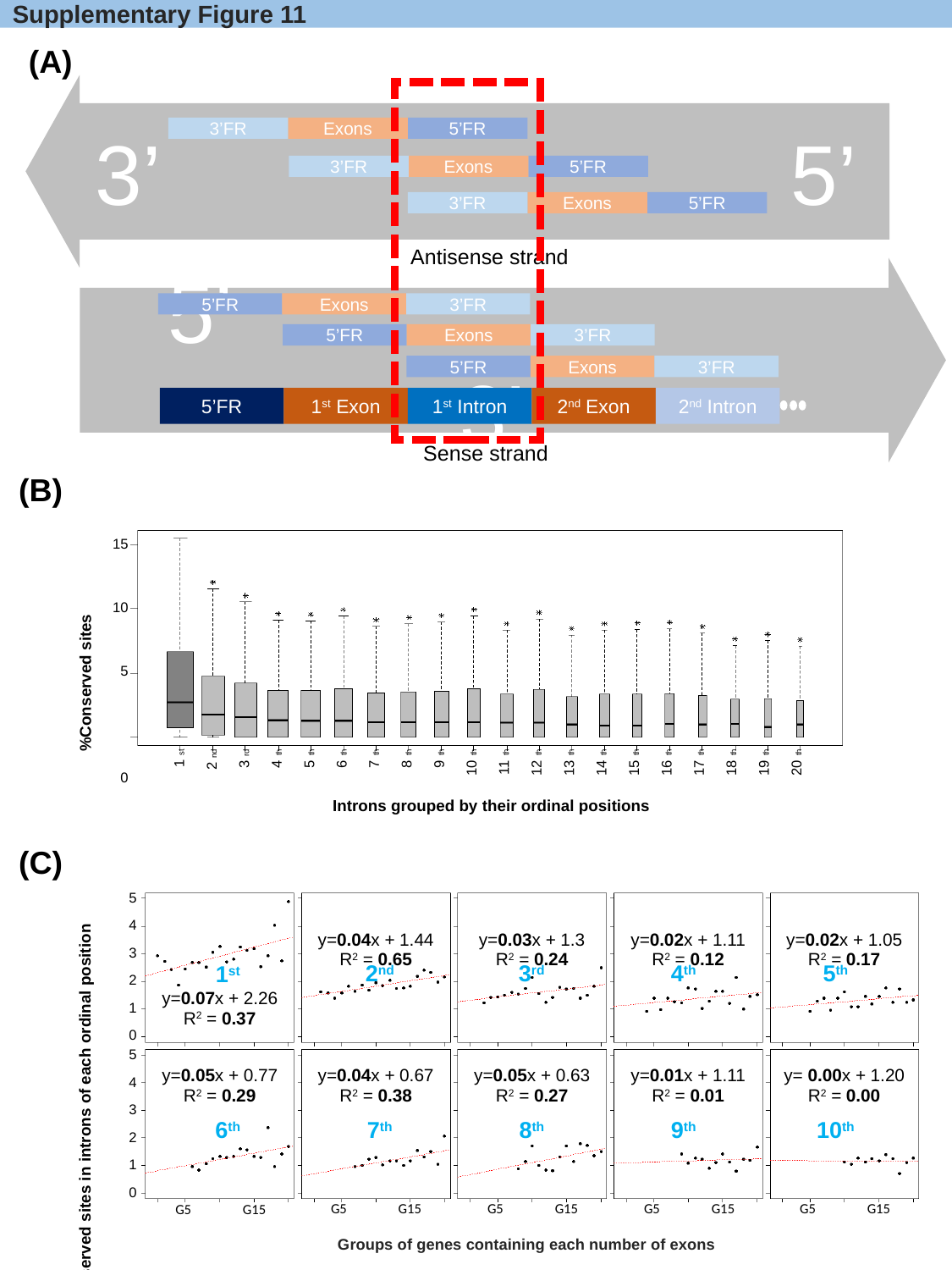

Supplementary Figure 11
(A)
3’ 5’
5’FR
Exons
3’FR
5’FR
Exons
3’FR
5’FR
Exons
3’FR
Antisense strand
 5’ 3’
5’FR
Exons
3’FR
5’FR
Exons
3’FR
5’FR
Exons
3’FR
5’FR
1st Exon
1st Intron
2nd Exon
2nd Intron
Sense strand
(B)
| %Conserved sites | 15 |
| --- | --- |
| | 10 |
| | 5 |
| | 0 |
| 1st | 2nd | 3rd | 4th | 5th | 6th | 7th | 8th | 9th | 10th | 11th | 12th | 13th | 14th | 15th | 16th | 17th | 18th | 19th | 20th |
| --- | --- | --- | --- | --- | --- | --- | --- | --- | --- | --- | --- | --- | --- | --- | --- | --- | --- | --- | --- |
| Introns grouped by their ordinal positions | | | | | | | | | | | | | | | | | | | |
(C)
| 5 |
| --- |
| 4 |
| 3 |
| 2 |
| 1 |
| 0 |
| y=0.07x + 2.26 R2 = 0.37 | y=0.04x + 1.44 R2 = 0.65 | y=0.03x + 1.3 R2 = 0.24 | y=0.02x + 1.11 R2 = 0.12 | y=0.02x + 1.05 R2 = 0.17 |
| --- | --- | --- | --- | --- |
| y=0.05x + 0.77 R2 = 0.29 | y=0.04x + 0.67 R2 = 0.38 | y=0.05x + 0.63 R2 = 0.27 | y=0.01x + 1.11 R2 = 0.01 | y= 0.00x + 1.20 R2 = 0.00 |
| 1st | 2nd | 3rd | 4th | 5th |
| --- | --- | --- | --- | --- |
| 6th | 7th | 8th | 9th | 10th |
| % Conserved sites in introns of each ordinal position |
| --- |
| 5 |
| --- |
| 4 |
| 3 |
| 2 |
| 1 |
| 0 |
| G5 | G15 |
| --- | --- |
| G5 | G15 |
| --- | --- |
| G5 | G15 |
| --- | --- |
| G5 | G15 |
| --- | --- |
| G5 | G15 |
| --- | --- |
Groups of genes containing each number of exons

## Slide 15
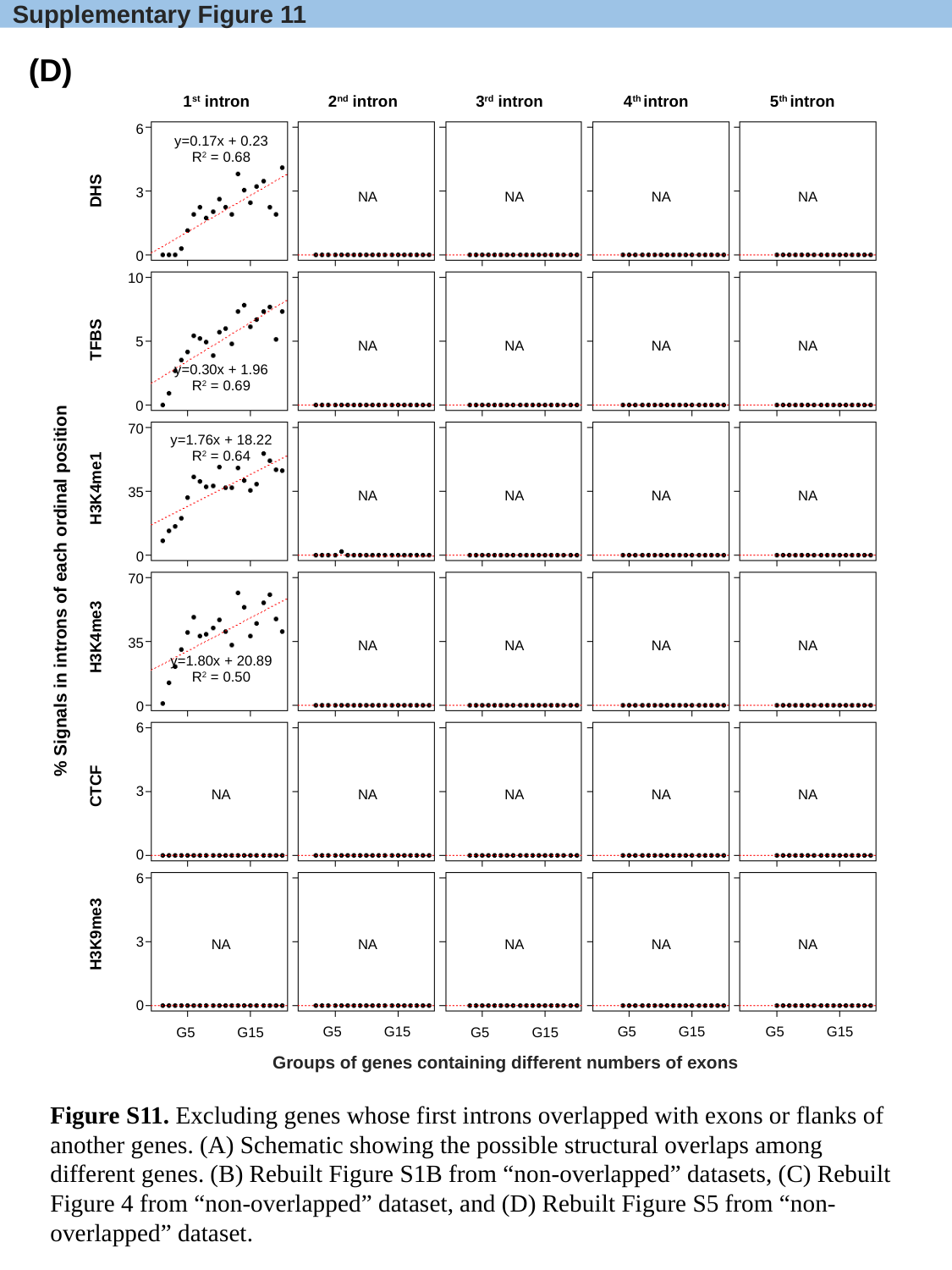

Supplementary Figure 11
(D)
| 1st intron | 2nd intron | 3rd intron | 4th intron | 5th intron |
| --- | --- | --- | --- | --- |
| DHS |
| --- |
| TFBS |
| H3K4me1 |
| H3K4me3 |
| CTCF |
| H3K9me3 |
| 6 |
| --- |
| 3 |
| 0 |
| y=0.17x + 0.23 R2 = 0.68 | NA | NA | NA | NA |
| --- | --- | --- | --- | --- |
| y=0.30x + 1.96 R2 = 0.69 | NA | NA | NA | NA |
| y=1.76x + 18.22 R2 = 0.64 | NA | NA | NA | NA |
| y=1.80x + 20.89 R2 = 0.50 | NA | NA | NA | NA |
| NA | NA | NA | NA | NA |
| NA | NA | NA | NA | NA |
| 10 |
| --- |
| 5 |
| 0 |
| % Signals in introns of each ordinal position |
| --- |
| 70 |
| --- |
| 35 |
| 0 |
| 70 |
| --- |
| 35 |
| 0 |
| 6 |
| --- |
| 3 |
| 0 |
| 6 |
| --- |
| 3 |
| 0 |
| G5 | G15 |
| --- | --- |
| G5 | G15 |
| --- | --- |
| G5 | G15 |
| --- | --- |
| G5 | G15 |
| --- | --- |
| G5 | G15 |
| --- | --- |
Groups of genes containing different numbers of exons
Figure S11. Excluding genes whose first introns overlapped with exons or flanks of another genes. (A) Schematic showing the possible structural overlaps among different genes. (B) Rebuilt Figure S1B from “non-overlapped” datasets, (C) Rebuilt Figure 4 from “non-overlapped” dataset, and (D) Rebuilt Figure S5 from “non-overlapped” dataset.

## Slide 16
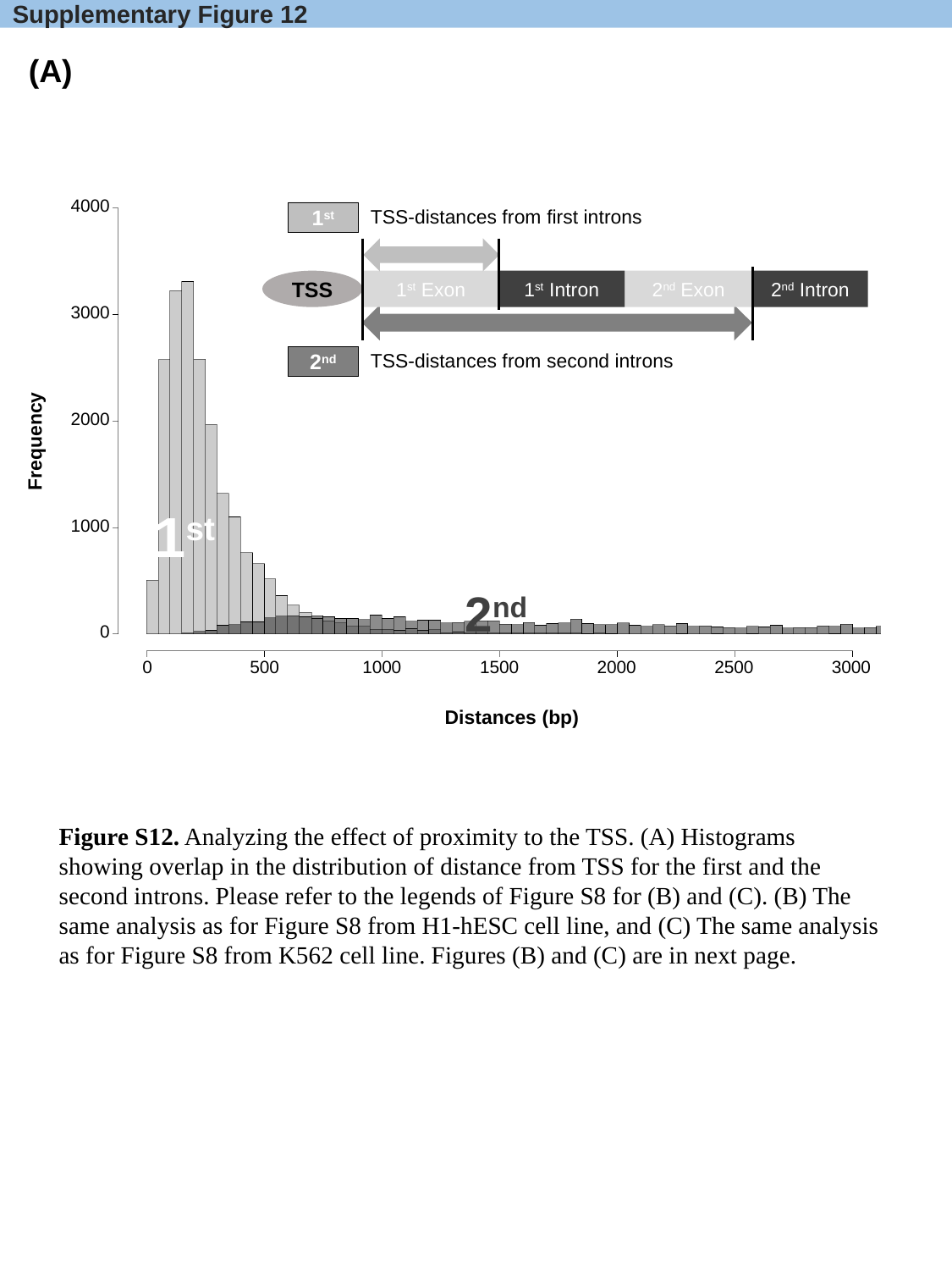

Supplementary Figure 12
(A)
| 4000 |
| --- |
| 3000 |
| 2000 |
| 1000 |
| 0 |
TSS-distances from first introns
1st
TSS
1st Exon
1st Intron
2nd Exon
2nd Intron
TSS-distances from second introns
2nd
Frequency
1st
2nd
| 0 | 500 | 1000 | 1500 | 2000 | 2500 | 3000 |
| --- | --- | --- | --- | --- | --- | --- |
Distances (bp)
Figure S12. Analyzing the effect of proximity to the TSS. (A) Histograms showing overlap in the distribution of distance from TSS for the first and the second introns. Please refer to the legends of Figure S8 for (B) and (C). (B) The same analysis as for Figure S8 from H1-hESC cell line, and (C) The same analysis as for Figure S8 from K562 cell line. Figures (B) and (C) are in next page.

## Slide 17
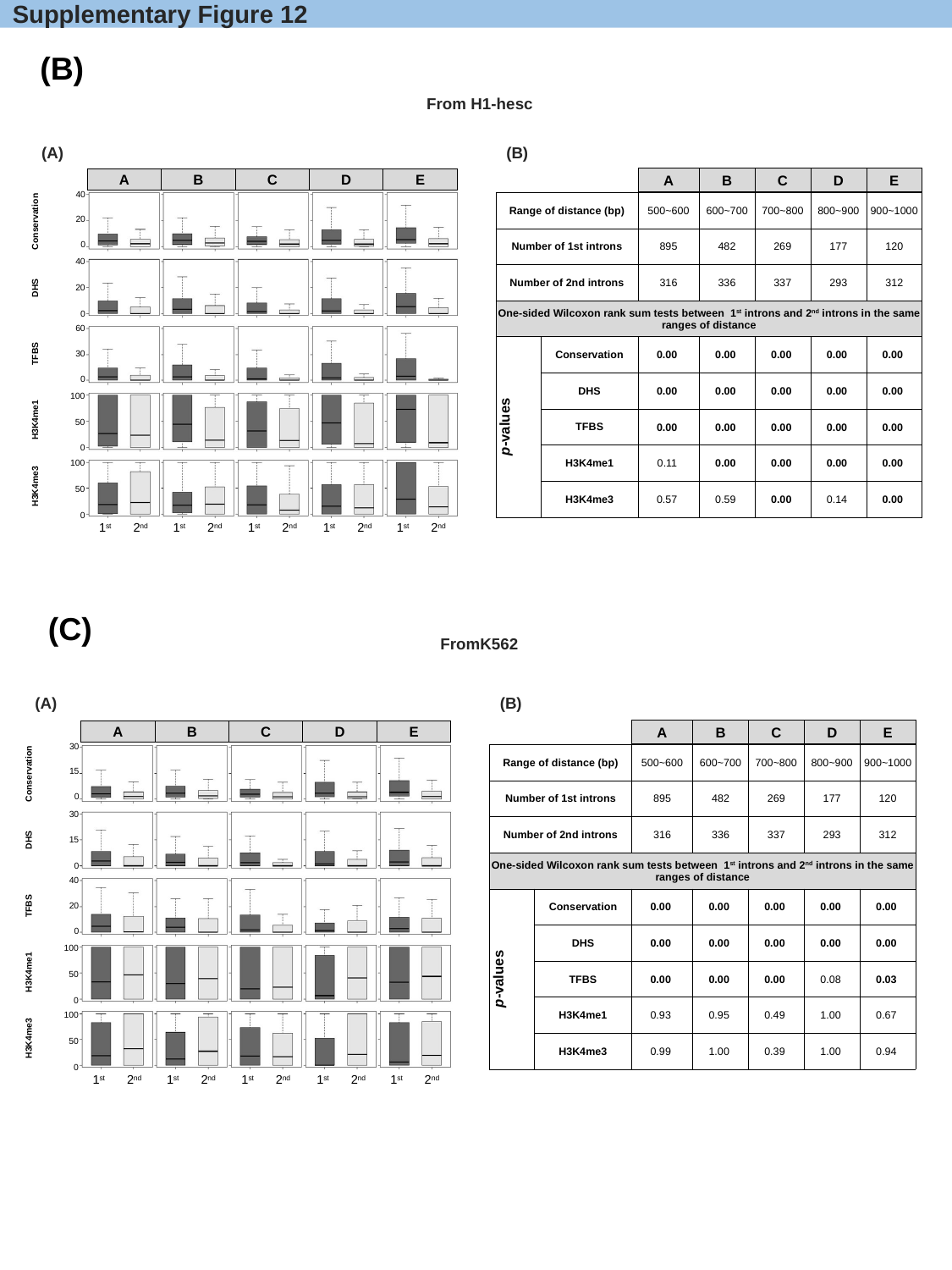

Supplementary Figure 12
(B)
From H1-hesc
(A)
(B)
| | | A | B | C | D | E |
| --- | --- | --- | --- | --- | --- | --- |
| Range of distance (bp) | | 500~600 | 600~700 | 700~800 | 800~900 | 900~1000 |
| Number of 1st introns | | 895 | 482 | 269 | 177 | 120 |
| Number of 2nd introns | | 316 | 336 | 337 | 293 | 312 |
| One-sided Wilcoxon rank sum tests between 1st introns and 2nd introns in the same ranges of distance | | | | | | |
| p-values | Conservation | 0.00 | 0.00 | 0.00 | 0.00 | 0.00 |
| | DHS | 0.00 | 0.00 | 0.00 | 0.00 | 0.00 |
| | TFBS | 0.00 | 0.00 | 0.00 | 0.00 | 0.00 |
| | H3K4me1 | 0.11 | 0.00 | 0.00 | 0.00 | 0.00 |
| | H3K4me3 | 0.57 | 0.59 | 0.00 | 0.14 | 0.00 |
| A | B | C | D | E |
| --- | --- | --- | --- | --- |
| 40 |
| --- |
| 20 |
| 0 |
| Conservation |
| --- |
| DHS |
| TFBS |
| H3K4me1 |
| H3K4me3 |
| 40 |
| --- |
| 20 |
| 0 |
| 60 |
| --- |
| 30 |
| 0 |
| 100 |
| --- |
| 50 |
| 0 |
| 100 |
| --- |
| 50 |
| 0 |
| 1st | 2nd |
| --- | --- |
| 1st | 2nd |
| --- | --- |
| 1st | 2nd |
| --- | --- |
| 1st | 2nd |
| --- | --- |
| 1st | 2nd |
| --- | --- |
(C)
FromK562
(A)
(B)
| | | A | B | C | D | E |
| --- | --- | --- | --- | --- | --- | --- |
| Range of distance (bp) | | 500~600 | 600~700 | 700~800 | 800~900 | 900~1000 |
| Number of 1st introns | | 895 | 482 | 269 | 177 | 120 |
| Number of 2nd introns | | 316 | 336 | 337 | 293 | 312 |
| One-sided Wilcoxon rank sum tests between 1st introns and 2nd introns in the same ranges of distance | | | | | | |
| p-values | Conservation | 0.00 | 0.00 | 0.00 | 0.00 | 0.00 |
| | DHS | 0.00 | 0.00 | 0.00 | 0.00 | 0.00 |
| | TFBS | 0.00 | 0.00 | 0.00 | 0.08 | 0.03 |
| | H3K4me1 | 0.93 | 0.95 | 0.49 | 1.00 | 0.67 |
| | H3K4me3 | 0.99 | 1.00 | 0.39 | 1.00 | 0.94 |
| A | B | C | D | E |
| --- | --- | --- | --- | --- |
| 30 |
| --- |
| 15 |
| 0 |
| Conservation |
| --- |
| DHS |
| TFBS |
| H3K4me1 |
| H3K4me3 |
| 30 |
| --- |
| 15 |
| 0 |
| 40 |
| --- |
| 20 |
| 0 |
| 100 |
| --- |
| 50 |
| 0 |
| 100 |
| --- |
| 50 |
| 0 |
| 1st | 2nd |
| --- | --- |
| 1st | 2nd |
| --- | --- |
| 1st | 2nd |
| --- | --- |
| 1st | 2nd |
| --- | --- |
| 1st | 2nd |
| --- | --- |
